# Relationship between the physical environment and different domains of physical activity in European adults: a systematic review

**DOI:** 10.1186/1471-2458-12-807

**Published:** 2012-09-19

**Authors:** Veerle Van Holle, Benedicte Deforche, Jelle Van Cauwenberg, Liesbet Goubert, Lea Maes, Nico Van de Weghe, Ilse De Bourdeaudhuij

**Affiliations:** 1 Department of Movement and Sports Sciences, Ghent University, Watersportlaan 2, B-9000, Ghent, Belgium; 2 Department of Human Biometry and Biomechanics, Vrije Universiteit Brussel, Pleinlaan 2, B-1050, Brussels, Belgium; 3 Department of Experimental Clinical and Health Psychology, Ghent University, Henri Dunantlaan 2, B-9000, Ghent, Belgium; 4 Department of Public Health, Ghent University, De Pintelaan 185 (Block A), B-9000, Ghent, Belgium; 5 Department of Geography, Ghent University, Krijgslaan 281 (S8), B-9000, Ghent, Belgium

**Keywords:** Domain-specific physical activity, Built environment, Continent-specific, Transportation

## Abstract

**Background:**

In the past decade, various reviews described the relationship between the physical environment and different physical activity (PA) domains. Yet, the majority of the current review evidence relies on North American/Australian studies, while only a small proportion of findings refer to European studies. Given some clear environmental differences across continents, this raises questions about the applicability of those results in European settings. This systematic review aimed at summarizing Europe-specific evidence on the relationship between the physical environment and different PA domains in adults.

**Methods:**

Seventy eligible papers were identified through systematic searches across six electronic databases. Included papers were observational studies assessing the relationship between several aspects of the physical environment and PA in European adults (18-65y). Summary scores were calculated to express the strength of the relationship between each environmental factor and different PA domains.

**Results:**

Convincing evidence on positive relationships with several PA domains was found for following environmental factors: walkability, access to shops/services/work and the composite factor environmental quality. Convincing evidence considering urbanization degree showed contradictory results, dependent on the observed PA domain. Transportation PA was more frequently related to the physical environment than recreational PA. Possible evidence for a positive relationship with transportation PA emerged for walking/cycling facilities, while a negative relationship was found for hilliness. Some environmental factors, such as access to recreational facilities, aesthetics, traffic- and crime-related safety were unrelated to different PA domains in Europe.

**Conclusions:**

Generally, findings from this review of European studies are in accordance with results from North American/Australian reviews and may contribute to a generalization of the relationship between the physical environment and PA. Nevertheless, the lack of associations found regarding access to recreational facilities, aesthetics and different forms of safety are likely to be Europe-specific findings and need to be considered when appropriate interventions are developed. More research assessing domain-specific relationships with several understudied environmental attributes (e.g., residential density) is needed.

## Background

Regular moderate-to-vigorous intensity physical activity (MVPA) contributes to several beneficial short- and long term health effects [1-3]. Unfortunately, about 31 percent (28% men, 34% women) of the global adult population is inadequately active to achieve health benefits [4]. To promote physical activity (PA) in the adult population, research investigating its possible underlying determinants and correlates is essential. While earlier research on this topic focused mainly on the contribution of personal determinants of PA behavior, social ecological models have been of growing interest during the last decade. These models put forward that domain-specific PA is influenced by multiple factors, which interact across different levels [5-7]. Of particular interest is the environmental level, including the physical environment. Davison and Lawson defined the physical environment as the objective and perceived characteristics of the physical context in which people spend their time (e.g., home, neighborhood), including aspects of urban design (e.g., presence of sidewalks), traffic density and speed, distance to and design of venues for PA (e.g., parks), crime and safety [8]. As physical environmental attributes are changeable and such changes can influence health-related behaviors such as PA, insight into physical environmental correlates of PA is crucial when developing interventions to promote PA.

At present, several reviews have summarized the available evidence on the relationship between the physical environment and different PA domains in adult populations [9-16]. Remarkably, the majority of discussed studies in these reviews were carried out in North American and Australian settings, while the proportion of studies conducted in other continents like Europe are more limited. Moreover, none of these reviews provided separate results for different geographical regions. Currently, it is not clear yet whether the results on environmental correlates of PA found in America or Australia are applicable to European countries, so further research is needed before transferring findings across continents. Since research on environmental correlates of food-intake shows that associations may well differ between countries [17], it is plausible that this is also true for environmental correlates of PA. Moreover, physical environmental attributes in Europe are likely to differ from an American or Australian context. For example, European urban streetscapes are characterized by a more compact structure, whereas most American cities are less dense due to sub-urbanization and existence of peripheral centers [18]. Because of these dissimilarities in density, average trip distances in Europe are shorter than in the US [19,20], which in turn can influence human behavior like active versus passive transport mode choices. Bassett and colleagues strengthen the assumption that also the behavior itself can be a continent-specific phenomenon, by showing that active transportation trips are much more common in Europe when compared to North America and Australia [21]. In addition to the above-mentioned geographical and behavioral differences, there has been a recent boost in European studies investigating physical environmental correlates of PA in adult populations, making it relevant to update the existing European literature on this topic.

In summary, there is uncertainty about the applicability of North American and Australian results on the relationship between the physical environment and adults’ PA in European settings. Additionally, European research in this field is growing and therefore, this systematic review aims to provide an overview of the available European evidence during the last decade. As PA can be subdivided into several domains (e.g., transportation, recreation) and particular environmental attributes may relate differently to specific PA domains [12,22], relationships between several physical environmental factors and specific PA domains will be investigated.

## Methods

### Search strategy

**Figure 1:**
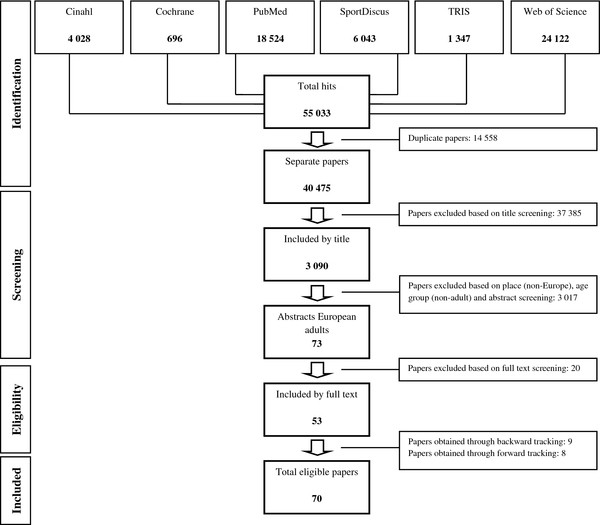
**Flow chart of the systematic literature search.** Included search terms: (determinant OR determinants OR correlate OR correlates OR influence OR influences OR association OR associations) AND (environment OR environmental OR physical OR built OR neighborhood OR neighbourhood OR facilities OR walkability OR aesthetics OR safety OR equipment) AND (physical activity OR physically active lifestyle OR leisure activities OR exercise OR exercising OR walk OR walking OR cycle OR cycling OR commute OR active commuting OR active transportation OR active travel) NOT (intervention OR comment OR disabled OR patients OR institutionalized).

Systematic searches were conducted across six electronic databases: Cinahl, Cochrane, PubMed, SportDiscus, TRIS and Web of Science. A two-stage search was conducted to identify eligible studies published between January 2000 and August 2011. In a first stage, the third author (JVC) screened databases until January 2010. In the second stage, an update of electronic database screening was conducted by the first author (VVH), who also performed all subsequent screening steps. Figure 1 provides an overview of the search protocol, according to the PRISMA statement [23] and specifies the used search terms*.* After excluding duplicates and making exclusions based on title and abstract, 73 papers remained. Twenty of these studies were excluded based on full text. Backward screening of the remaining 53 papers’ reference lists and forward screening of citations yielded 17 more papers, resulting in a total amount of 70 eligible papers [24-93] for this review. During the entire screening process, eligibility of doubtful publications was discussed with the second author (BD) until consensus was reached.

### Eligibility criteria

During database screening, following inclusion criteria were applied: suitable papers were restricted to English-written observational studies on European adult samples (mean age of the study population between 18 and 65y, or – in case no mean age was provided – an age range restriction from 18-65y). Eligible publications had to be cross-sectional or longitudinal studies, investigating the relationship between objective or subjective measures of PA and the physical environment. Exclusion criteria were set as follows: studies describing exclusively non-European samples and/or populations outside the specified age range were not eligible. Papers were also excluded when they considered exclusively physical environmental measures, or PA, respectively. Furthermore, studies focusing only on occupational and/or household PA as dependent variable were excluded, since these behaviors are bounded to very specific contexts (i.e., the workplace and home residence) and, consequently, are less susceptible to changes in physical environmental attributes of the residential neighborhood. Concerning the independent variable, studies that only focused on the socio-cultural, economic or policy environment were excluded. From a study design perspective, qualitative reports, interventions, experiments, case studies and experts’ opinions were not eligible. At last, studies focusing on disabled, unhealthy, overweight, obese or pregnant participants were excluded.

### Selection of the variables

Included dependent variables were measures of 1) total PA, 2) leisure-time PA (LTPA), 3) total walking and/or cycling, 4) recreational walking and/or cycling, 5) active transportation in general, 6) transportation walking and 7) transportation cycling. Physical environmental characteristics were classified according to the categories applied in the valid and reliable Neighborhood Environment Walkability Scale (NEWS questionnaire, [94-96]), which is the most internationally used questionnaire to assess perceptions of the environmental correlates of PA [97,98]. Retained independent variables were 1) walkability and its three key elements: 2) residential density, 3) land use mix diversity, and 4) street connectivity. Further included independent variables were 5) access to shops/services/workplace, 6) access to public transport, 7) access to recreational facilities, including greenery and places or facilities for PA, 8) quality and presence of walking and cycling facilities, 9) general safety, 10) traffic safety, 11) safety from crime and 12) aesthetic features. In addition to the NEWS categories, three other environmental attributes were included as independent variables. As worldwide studies already revealed that urban–rural differences are associated with variations in PA [99], “degree of urbanization” was added as a 13^th^ variable, often expressed as a measure of a region’s population density or the size of the municipality. Throughout the screening process, the 14^th^ variable “hilliness” and 15^th^ variable “quality of the environment”, a composite environmental measure assessing general activity-friendliness, were identified as important variables in the research domain. Studies were included if they provided results on relationships between at least one of the above-mentioned dependent and at least one of the independent variables.

### Data extraction

Next, data extraction tables were constructed for each separate PA category mentioned above. Study results were coded as significant positive “+”, significant negative “-”, or insignificant “0” relationships. If both univariate and multivariate results were provided, the univariate results were considered, in order to keep comparability between different studies as high as possible. For the same reason and when available, study results controlling for the least variables were retained [100]. When analyses were conducted separately for male and female participants, respectively ”M” or ”F” was indicated in superscript. If analyses were conducted for different subgroups in a study (e.g. low vs high SES or separate countries in a multi-country study), superscript numbers were added. If analyses were done for different time periods, superscripts “I” and “II” were added. Finally, as outcomes based upon objective and perceived measurements of both PA [101] and the physical environment [102] can differ, a distinction was made between these measurement methods: regular font was used when both PA and the physical environment were measured subjectively. Objective measures of PA and the physical environment were indicated by using italics and bold font, respectively. A more detailed description of all measures per individual study is accessible in “Additional file 1”.

### Coding of the evidence

Further classification of the evidence was based upon criteria provided in the review of Wendel-Vos and colleagues [14]. In specific, the number of times an environmental factor was significantly related to a PA domain was divided by the total amount of records on this relationship. When associations in one direction were found in more than 50% of all records, this was regarded as convincing evidence, summary coded “+” for a positive association and “-” for a negative. However, in case simultaneously at least 25% of all records reported results in the opposite direction, this was regarded as only possible evidence, summary coded “(+)” or “(−)” for a possible positive or negative association, respectively. Summary codes for possible evidence were also applied if an association was found in 40-50% of all records. Associations found in less than 40% of all records, or in 40-50% of all records in one direction with at least 25% in the opposite, was regarded as no evidence, summary coded “0”. Double signed summary codes were applied when convincing positive “++”, convincing negative “−−”, possible positive “(++)”, possible negative “(−−)” or no “00” associations were present in at least four independent samples, and this was regarded as strong evidence. Yet, all aforementioned coding only counted when a relationship was investigated in at least three independent samples, otherwise evidence was considered as not applicable, coded “N/A”. An overview of the summary coding is provided in Table 1.

**Table 1:** Criteria for summary coding of the evidence

**Percentages of records supporting association**^**1**^	**Summary code**^**2**^	**Description**
0-39% associated	0	Evidence unrelated
40-50% associated in one direction and ≥25% in the opposite	0	Evidence unrelated
40-50% associated in one direction and < 25% in the opposite	(+);(−)	Possible evidence for a positive/negative relationship
51-100% associated in one direction and ≥25% in the opposite	(+);(−)	Possible evidence for a positive/negative relationship
51-100% associated in one direction and < 25% in the opposite	+; -	Convincing evidence for a positive/negative relationship

^1^ Only valid when relationship was investigated in at least three independent samples, otherwise evidence was regarded "not applicable" (coded N/A).

^2^ Double signed summary codes are applied when convincing positive “++”, convincing negative “--”, possible positive “(++)”, possible negative “(−−)” or no “00” associations were present in at least four independent samples.

## Results

### Study characteristics

Across the 70 retained papers, data gathered in 66 unique samples across 27 European countries was available. As depicted in Table 2, the largest part of studies were conducted in the United Kingdom, Belgium and The Netherlands, respectively covering 19, 16 and 13 publications. Twenty-one studies calculated split results for men and women and four studies analyzed data for separate subgroups. Only one study [43] provided longitudinal data. Regarding PA measurement methods, six studies used objective data, compared to 59 studies using subjective data. Another five studies used both objective and subjective PA measurement methods. For environmental measurement methods, the distribution was more balanced: 31 studies used only objective data, 28 studies only subjective and 11 studies combined both. Total PA was the most studied PA variable, measured in 34 studies, while total cycling was the least studied, with only two studies that assessed it as a separate variable. The most studied environmental variable was access to recreation facilities, which was measured in 31 studies, and the least studied environmental variable, appearing in three studies, was hilliness. A complete overview of sample sizes, mean ages, study designs and measurement methods is shown in Table 3.

**Table 2:** Overview of the European countries' distribution across studies

**Country**	**Reference number**	**Total**
Bosnia-Herzegovina	45*	1
Estonia	45*	1
Georgia	45*	1
Ireland	69*	1
Luxembourg	69*	1
Poland	51	1
Turkey	45*	1
Ukraine	45*	1
Croatia	45*, 56	2
Denmark	69*, 81	2
Greece	66, 69*	2
Lithuania	37*, 55*, 73*	3
Austria	69*, 77, 79, 80	4
Czech Republic	36, 42, 45*, 74	4
Hungary	37*, 45*, 55*, 73*	4
Slovakia	37*, 45*, 55*, 73*	4
Finland	24*, 37*, 68*, 69*, 76*	5
Switzerland	37*, 55*, 68*, 73*, 76*	5
France	24*, 27, 55*, 69*, 73*	5
Italy	24*, 30, 55*, 69*, 73*	5
Sweden	25, 26, 28, 44, 69*, 78	6
Germany	24*, 37*, 55*, 68*, 69*, 73*, 76*	7
Portugal	37*, 55*, 69*, 70, 71, 72, 73*	7
Spain	24*, 29, 45*, 57, 65, 68*, 69*, 76*	8
The Netherlands	24*, 38, 43, 49, 50, 52, 53, 68*, 69*, 76*, 88, 92, 93	13
Belgium	33, 34, 35, 59, 68*, 69*, 76*, 82, 83, 84, 85, 86, 87, 89, 90, 91	16
UK	24*, 31, 32, 39, 40, 41, 46, 47, 48, 54, 58, 60, 61, 62, 63, 64, 66, 69*, 75	19

*Country was involved as part of a multi-country study.

**Table 3:** Categorization of samples by size, mean age, design, environmental and physical activity variables

	**Reference number**	**Total**
**Sample size**		
<100	36	1
100 – 199	44^1^, 82	2
200 – 299	33^M^, 33^F^, 34^1^, 34^2^, 74^M^	5
300 – 499	35, 44^2^, 59, 60, 61, 69^14^, 74^F^, 77^M^, 84, 86	10
500 – 999	31, 38, 53^M^, 53^F^, 55^M^, 57, 62^M^, 62^F^, 69^12^, 77^F^, 79, 80	12
1000 – 2999	25, 26, 30, 39^M^, 39^F^, 55^F^, 58, 68^M^, 68^F^, 69^1^, 69^2^, 69^3^, 69^4^, 69^5^, 69^6^, 69^7^, 69^8^, 69^9^, 69^10^, 69^11^, 69^12^, 69^13^, 69^15^, 69^16^, 69^17^, 73^M^, 73^F^, 78, 83, 85, 93	31
3000 – 4999	24, 27^M^, 27^F^, 39, 42^M^, 47, 49, 51^M^, 51^F^, 52, 56^M^, 56^F^, 66, 71^M^, 71^F^, 72^M^, 72^F^, 76, 87, 88^1^, 88^2^	21
5000 – 9999	29^M^, 29^F^, 32, 37, 40^M^, 40^F^, 41^M^, 41^F^, 42^F^, 43^MI^, 43^MII^, 43^FI^, 43^FII^, 48, 54, 64, 70, 73, 75	19
≥ 10000	28, 45^M^, 45^F^, 46, 50, 63, 65^M^, 65^F^, 67, 81, 89, 90, 91, 92	14
**Mean age (years)**		
18.0 – 29.9	57, 79	2
30.0 – 39.9	34, 36, 51, 71, 72, 74, 75, 77, 80, 90, 91	11
40.0 – 49.9	24, 25, 26, 28, 29, 33, 35, 38, 39, 42, 44, 45, 46, 49, 52, 53, 55, 58, 59, 65, 66, 67, 68, 73, 76, 78, 81, 82, 83, 84, 85, 86, 87, 88, 89, 93	36
50.0 – 59.9	27, 32, 48, 60, 61	5
60.0 – 64.9	40, 41, 47, 62	4
only provision of age range	30, 31, 37, 43, 50, 54, 56, 63, 64, 69, 70, 93	12
**Study design**		
Cross-sectional	24, 25, 26, 27, 28, 29, 30, 31, 32, 33, 34, 35, 36, 37, 38, 39, 40, 41, 42, 44, 45, 46, 47, 48, 49, 50, 51, 52, 53, 54, 55, 56, 57, 58, 59, 60, 61, 62, 63, 64, 65, 66, 67, 68, 69, 70, 71, 72, 73, 74, 75, 76, 77, 78, 79, 80, 81, 82, 83, 84, 85, 86, 87, 88, 89, 90, 91, 92, 93	69
Longitudinal	43	1
**Measurement environment**		
Objective	27, 28, 30, 32, 36, 37, 40, 41, 43, 45, 47, 51, 52, 53, 56, 65, 66, 70, 78, 79, 80, 82, 83, 84, 86, 87, 88, 89, 90, 91, 92	31
Subjective	24, 25, 26, 33, 34, 35, 38, 39, 42, 44, 46, 49, 50, 59, 60, 63, 67, 68, 69, 71, 72, 73, 74, 75, 76, 77, 85, 93	28
Both	29, 31, 48, 54, 55, 57, 58, 61, 62, 64, 81	11
**Measurement PA**		
Objective	36, 64, 74, 75, 90, 91	6
Subjective	24, 25, 26, 27, 28, 29, 30, 31, 32, 33, 34, 35, 37, 38, 40, 39, 41, 42, 43, 44, 45, 46, 47, 48, 49, 50, 51, 52, 53, 54, 55, 56, 57, 58, 59, 60, 61, 62, 63, 65, 66, 67, 68, 69, 70, 71, 72, 73, 76, 77, 78, 79, 80, 81, 87, 88, 89, 92, 93	59
Both	82, 83, 84,85, 86	5
**Environmental variables**		
Walkability	36, 59, 61, 78, 82, 83, 85, 86, 87	9
Residential density	32, 33, 34, 53, 55, 87	6
Land use mix diversity	32, 33, 34, 62, 80	5
Street connectivity	32, 33, 34, 62, 85	5
Access to shops/services/work	31, 33, 34, 35, 38, 39, 46, 53, 54, 57, 58, 62, 63, 64, 67, 70, 74, 77, 85, 88, 90, 91	22
Access to public transport	33, 34, 35, 46, 50, 53, 57, 93	8
Access to recr. facilities	24, 28, 29, 30, 32, 33, 34, 40, 39, 44, 46, 47, 48, 49, 52, 54, 58, 60, 61, 62, 65, 67, 68, 69, 70, 73, 74, 76, 81, 85, 91, 92	32
Walking/cycling facilities	26, 33, 34, 35, 57, 58, 62, 64, 74, 80, 85, 90, 91	13
Safety	39, 46, 48, 49, 54, 55, 63, 71, 72, 73, 74, 80, 85	13
Traffic-related safety	26, 31, 32, 33, 34, 35, 40, 39, 41, 46, 55, 58, 62, 64, 70, 79, 80, 85, 90, 91	20
Crime-related safety	26, 31, 33, 34, 35, 40, 46, 49, 58, 62, 63, 67, 70, 75, 79, 85, 88, 90, 91	19
Aesthetics	29, 31, 33, 34, 69, 39, 49, 54, 55, 58, 63, 73, 74, 79, 80, 85, 88, 91	18
Hilliness	64, 80, 91	3
Urbanization	25, 26, 27, 29, 35, 42, 43, 45, 51, 54, 56, 62, 64, 66, 67, 70, 81, 84, 89, 90, 91	21
Quality of the environment	29, 54, 61, 71, 72, 74, 77	7
**PA variables**		
Total PA	24, 25, 26, 28, 30, 31, 32, 33, 34, 36, 37, 42, 44, 45, 46, 48, 52, 56, 57, 58, 61, 67, 68, 69, 72, 74, 76, 78, 82, 83, 84, 85, 86, 87	34
Leisure-time PA (LTPA)	27, 29, 34, 40, 42, 43, 47, 49, 52, 55, 60, 61, 65, 66, 67, 70, 70, 73, 75, 77, 81, 84, 85, 86, 88, 89	26
Total walking	26, 33, 39, 42, 54, 61, 67, 71, 92, 93	10
Total cycling	92, 93	2
Recreational walking	34, 40, 52, 63, 78, 82, 83, 84, 85, 86, 92	11
Recreational cycling	40, 41, 52, 82, 84, 92	6
Active transportation	34, 51, 57, 58, 62, 88	6
Walking for transportation	50, 52, 63, 77, 78, 82, 83, 84, 85, 86, 92	11
Cycling for transportation	35, 38, 41, 50, 52, 53, 59, 64, 77, 79, 80, 82, 83, 84, 85, 86, 90, 91, 92	19

M = male subgroup; F = female subgroup; I = subgroup 1st measurement period; II = subgroup 2nd measurement period; superscript numbers indicate subgroups based on other classification criteria (e.g., country).

### Physical environment and the relationship with total physical activity

Thirty-four studies assessed relationships between aspects of the physical environment and measures of total PA. Summary results considering this relationship are depicted in Table 4. Convincing evidence for a positive relationship with total PA was found for the factors walkability and quality of the environment, with a strong relationship for walkability (results of at least four independent samples underpin the relationship). For urbanization degree, there was convincing evidence for a negative relationship, which means that people living in less urbanized areas tended to be more physically active. Further, possible evidence for a positive relationship emerged between total PA and access to recreation facilities. All other environmental factors were unrelated to total PA.

**Table 4:** Summary results of evidence on the relationship environmental factors and total PA

**Environm. variables**	**Positive association**	**Negative association**	**No association**	A*	B*	C*	D*
Walkability	***36***, 61, **78**, ***82, 82***^***2***^***, 83***, *85*, ***86***, **87**		***82***^***1***^	8	9/10	90	++
Residential density	**87**		**32**, 33^M^, 33^M^, 33^F^, 33^F^, 34, 34	4	1/8	13	0
Land use mix diversity	34		**32**, 33^M^, 33^M^, 33^F^, 33^F^, 34	3	1/7	14	0
Street connectivity	**32**, **32**, **32**		33^M^, 33^M^, 33^F^, 33^F^, 34, 34, *85*	4	3/10	30	0
Access to shops/services/work	31, 31, 31, 31, 31, 33^F^, 67, *74*^*F*^	8	33^M^, 33^M^, 33^F^, 34, 34, 46, **57**, **58**, 58, 58, 67, *74*^*M*^*, 74*^*M*^, *74*^*F*^, *82, 82, 85*	9	8/26	31	00
Access to public transport			33^M^, 33^M^, 33^F^, 33^F^, 34, 34, 46, 57	4	0/8	0	00
Access to recreation facilities	24, **28**, **28**, **30**, **30**,33^M^, 33^F^, 44^1^, 44^2^, 46, 48^1^, 48^2^, **61**, 67, 68^M^, 68^F^, 69^1^, 69^2^, 69^3^, 69^4^, 69^5^, 69^6^, 69^7^, 69^9^, 69^10^, 69^11^, 69^12^, 69^14^, 69^15^, 69^16^, 76		**32**, 33^M^, 33^F^, 34, 34, **48**^**1**^, **48**^**1**^, **48**^**1**^, **48**^**1**^, **48**^**1**^, **48**^**1**^, **48**^**2**^, **48**^**2**^, **48**^**2**^, **48**^**2**^, **48**^**2**^, **48**^**2**^, **52**, **52**, 58, **61**, **61**, 69^8^, 69^13^, 69^17^, *74*^*M*^, *74*^*M*^, *74*^*F*^, *74*^*F*^, *85*	17	31/61	51	(++)
Walking/cycling facilities	26		26, 33^M^, 33^M^, 33^M^, 33^M^, 33^F^, 33^F^, 33^F^, 33^F^, 34, 34, 34, 34, 57, 58, *74*^*M*^, *74*^*F*^, *85*, *85*	7	1/20	5	00
Safety	46, 46, 48^1^, 48^2^		72^M^, 72^M^, 72^F^, 72^F^, *74*^*M*^, *74*^*F*^, *85*	6	4/11	36	0
Traffic-related safety	26, **31**, **31**	**32**, **32**, 46, 58	26, 33^M^, 33^M^, 33^F^, 33^F^, 34, 34, 46, 58, *85*	7	4/17	24	00
Crime-related safety	26, **31**	46, 67	26, 33^M^, 33^M^, 33^F^, 33^F^, 34, 34, 46, 46, 58, 67, *85*	7	2/16	13	00
Aesthetics	31, **37**, **37**, *74*^*M*^		33^M^, 33^M^, 33^F^, 33^F^, 34, 34, 58, *74*^*F*^, *85*	7	4/13	31	00
Urbanization		25, 26, 42^M^, 42^M^, 42^F^, 42^F^**45**^**M**^, **45**^**F**^, **56**^**M**^, **56**^**F**^	25, 26, 67, ***84***	7	10/14	71	--
Quality of environment	61, 72^F^, 72^F^, *74*^*M*^, *74*^*F*^		72^M^, 72^M^	3	5/7	71	+

* A = n° of independent studies; B = n° of associated records divided by all records; C = % of evidence; D = summary code.

Regular vs italics font = subjective vs objective PA measures; regular vs bold font = subjective vs objective environmental measures.

^M^ = specific results for males; ^F^ = specific results for females; ^1^ = specific results for 1^st^ subgroup; ^2^ = specific results for 2^nd^ subgroup; ^n^ = specific results for n^th^ subgroup.

### Physical environment and the relationship with leisure-time physical activity

The relationship between the physical environment and LTPA was examined in 26 studies, of which summary results are presented in Table 5. There was only possible evidence for a positive relationship between LTPA and quality of the environment, whereas the factors walkability, access to shops/services/work, access to recreation facilities, general safety, traffic- and crime-related safety, aesthetics and urbanization were all unrelated to LTPA. Summary results could not be calculated for the three separate components of walkability, access to public transport and walking/cycling facilities because too few studies investigated the relationship of these environmental factors with LTPA.

**Table 5:** Summary results of evidence on the relationship environmental factors and leisure-time PA (LTPA)

**Environm. variables**	**Positive association**	**Negative association**	**No association**	A*	B*	C*	D*
Walkability	61		85, 85, **86**	3	1/4	25	0
Residential density			34, 34, **55**^**M**^, **55**^**M**^,**55**^**F**^, **55**^**F**^	2	N/A	N/A	N/A
Land use mix diversity	34		34	1	N/A	N/A	N/A
Street connectivity			34, 34, 85, 85	2	N/A	N/A	N/A
Access to shops/services/work	**70**, **88**		34, 34, 67, 67, **70**, **70**, **70**, 77, 77, 85, 85	5	2/13	15	00
Access to public transport			34, 34	1	N/A	N/A	N/A
Access to recreation facilities	29^M^, 29^F^, **40**^**F**^, **52**, 60, 60, 67, **70**, 81, 81, 85	60, 60	34, 34, **40**^**M**^, **40**^**M**^, **40**^**M**^, **40**^**M**^, **40**^**M**^, **40**^**F**^, **40**^**F**^, **40**^**F**^, **40**^**F**^, **47**, 49, **52**, **52**, **52**, 60, 60, **61**, **61**, **61**, **65**^**M**^, **65**^**M**^, **65**^**F**^, **65**^**F**^, **70**, **70**, **70**, 73, 73, 73^M^, 73^M^, 73^F^, 73^F^, 85	14	11/48	23	00
Walking/cycling facilities			34, 34, 34, 34, 85, 85, 85, 85	2	N/A	N/A	N/A
Safety	49	73, 73^M^, 73^F^, 73^F^	55^M^, 55^M^, 55^F^, 55^F^, 73, 73^M^, 85, 85	3	4/13	31	0
Traffic-related safety			34, 34, **55**^**M**^, **55**^**M**^, **55**^**F**^, **55**^**F**^,**70**, **70**, 85, 85	3	0/10	0	0
Crime-related safety	**70**, *75*		34, 34, 49, 67, 67, **70**, 85, 85, **88**, **88**	6	2/12	17	00
Aesthetics	49, **55**^**F**^, 73, 73, 73^M^, **88**, **88**		29^M^, 29^M^, 29^M^, 29^M^, 29^F^, 29^F^, 29^F^, 29^F^, 34, 34, **55**^**M**^, **55**^**M**^, **55**^**F**^, 73^M^, 73^F^, 73^F^, 85, 85, **88**	6	7/26	27	00
Urbanization	**29**^**M**^, **40**, **81**, **89**, **89**^**M**^	**27**^**F**^, 42^M^, 42^F^ , **66**	**27**^**M**^, **29**^**F**^, **43**^**MI**^**, 43**^**MII**^**, 43**^**FI**^**, 43**^**FII**^, 67, **70**, **70**, ***84***, ***84***, **89**, **89**, **89**, **89**^**M**^, **89**^**M**^, **89**^**M**^, **89**^**F**^, **89**^**F**^, **89**^**F**^**, 89**^**F**^	10	5/30	17	00
Quality of environment	77^M^, 77^F^		29^M^, 29^F^,61	3	2/5	40	(+)

* A = n° of independent studies; B = n° of associated records divided by all records; C = % of evidence; D = summary code.

Regular vs italics font = subjective vs objective PA measures; regular vs bold font = subjective vs objective environmental measures.

^M^ = specific results for males; ^F^ = specific results for females; ^I^ = specific results for 1^st^ measurement period; ^II^ = specific results for 2^nd^ measurement period.

### Physical environment and the relationship with total walking and cycling

Summary results for the studies investigating the relationship between physical environmental factors and total walking and cycling are shown in Table 6. Out of the 10 studies, eight [26,33,39,42,54,61,67,71] studied only walking and two [92,93] studied both walking and cycling as separate variables. Possible evidence for a positive relationship was found between urbanization degree and total walking, which means that people living in more urbanized areas possibly walked more than people living in less urbanized regions. Summary calculations resulted in no association of total walking and cycling with access to shops/services/work, access to recreation facilities, general safety, traffic- and crime-related safety and aesthetics. Study results were not applicable for the relationship between the remaining physical environmental factors and total walking and cycling due to a lack of sufficient individual studies in these categories.

**Table 6:** Summary results of evidence on the relationship environmental factors and total walking/cycling

**Environm. variables**	**Positive association**	**Negative association**	**No association**	A*	B*	C*	D*
Walkability			61	1	N/A	N/A	N/A
Residential density			33^M^, 33^F^	1	N/A	N/A	N/A
Land use mix diversity	33^F^		33^M^	1	N/A	N/A	N/A
Street connectivity			33^M^, 33^F^	1	N/A	N/A	N/A
Access to shops/services/work	54, 54, 54, 67		33^M^, 33^F^, 39^M^, 39^M^, 39^F^, 39^F^ 54, 54, 54, 54, 54, 67	4	4/16	25	00
Access to public transport	33^F^		33^M^, 93, 93	2	N/A	N/A	N/A
Access to recreation facilities	54, 54, **61**, **92**		33^M^, 33^F^, 39^M^, 39^M^, 39^M^, 39^M^, 39^F^, 39^F^, 39^F^, 39^F^, **61**, **61**, 67, **92**, **92**, **92**, **92**, **92**, **92**, **92**, **92**, **92**, **92**, **92**, **92**, **92**, **92**, **92**, **92**, **92**, **92**, **92**	6	4/36	11	00
Walking/cycling facilities	26, 33^M^		33^M^, 33^F^, 33^F^	2	N/A	N/A	N/A
Safety	54, 71		39^M^, 39^M^, 39^M^, 39^M^, 39^F^, 39^F^, 39^F^, 39^F^, 71	3	2/11	18	0
Traffic-related safety			26, 33^M^, 33^F^, 39^M^, 39^M^, 39^F^, 39^F^	3	0/7	0	0
Crime-related safety			26, 33^M^, 33^F^, 67, 67	3	0/5	0	0
Aesthetics	54		33^M^, 33^F^, 39^M^, 39^M^, 39^F^, 39^F^, 54, 54, 54	3	1/10	10	0
Urbanization	26, **54**		42^M^, 42^F^, 67	3	2/5	40	(+)
Quality of environment	71	71	61	2	N/A	N/A	N/A

* A = n° of independent studies; B = n° of associated records divided by all records; C = % of evidence; D = summary code.

Regular vs italics font = subjective vs objective PA measures; regular vs bold font = subjective vs objective environmental measures.

^M^ = specific results for males; ^F^ = specific results for females.

### Physical environment and the relationship with recreational walking and cycling

Twelve studies, presented in Table 7, assessed relationships between the physical environment and recreational walking and cycling. Six studies [34,63,78,83,85,86] investigated only walking, one study [41] focused on only cycling and five studies [40,52,82,84,92] measured both. Convincing evidence for a positive relationship with recreational walking and cycling emerged for traffic-related safety, which means that people living in less trafficked (and thus potentially safer) areas walked or cycled more for recreation. Further, evidence showed no association of recreational walking and cycling with walkability, access to shops/services/work, access to recreation facilities, crime-related safety and aesthetics. Results considering the remaining factors were not applicable due to a lack of sufficient studies in each category.

**Table 7:** Summary results of evidence on the relationship environmental factors and recreational walking/cycling

**Environm. variables**	**Positive association**	**Negative association**	**No association**	A*	B*	C*	D*
Walkability	**78**, **83,** 85		**82**, **82, 82**^**1**^**, 82**^**2**^**, 86**	5	3/8	38	0
Residential density			34, 34	1	N/A	N/A	N/A
Land use mix diversity	34		34	1	N/A	N/A	N/A
Street connectivity		85	34, 34	2	N/A	N/A	N/A
Access to shops/services/work			34, 34, 63, 85	3	0/4	0	0
Access to public transport			34, 34	1	N/A	N/A	N/A
Access to recreation facilities	**92**, **92**, **92**	**52**, **52**, **52**	34, 34, **40**^**M**^, **40**^**M**^, **40**^**M**^, **40**^**M**^, **40**^**F**^, **40**^**F**^, **40**^**F**^, **40**^**F**^, **52**, 85, **92**, **92**, **92**, **92**, **92**, **92**, **92**, **92**, **92**, **92**, **92**, **92**, **92**, **92**, **92**, **92**, **92**	5	3/35	9	00
Walking/cycling facilities	34		34, 34, 34, 85, 85	2	N/A	N/A	N/A
Safety	63		85	2	N/A	N/A	N/A
Traffic-related safety	**40**^**M**^, **40**^**F**^, **41**^**M**^, **41**^**F**^		34, 34, 85	4	4/7	57	+
Crime-related safety			34, 34, **40**^**M**^, **40**^**F**^, 63, 85	4	0/6	0	00
Aesthetics	63		34, 34, 63, 63, 85	3	1/6	17	0
Urbanization	**84**		**84, 84, 84**	1	N/A	N/A	N/A

* A = n° of independent studies; B = n° of associated records divided by all records; C = % of evidence; D = summary code.

Regular vs italics font = subjective vs objective PA measures; regular vs bold font = subjective vs objective environmental measures.

^M^ = specific results for males; ^F^ = specific results for females; ^1^ = specific results for 1^st^ subgroup; ^2^ = specific results for 2^nd^ subgroup.

### Physical environment and the relationship with general active transportation

Six studies investigated relationships of the physical environment with general active commuting. Results of the summary calculations are depicted in Table 8. Convincing evidence for a positive relationship was found between active transportation and access to shops/services/work and possible evidence for a positive relationship with active transportation emerged for walking/cycling facilities. Access to recreation facilities, traffic- and crime-related safety and aesthetics were all unrelated to active transportation. Summary results of relationships for general active transportation with other environmental factors were not applicable.

**Table 8:** Summary results of evidence on the relationship environmental factors and general active transportation

**Environm. variables**	**Positive association**	**Negative association**	**No association**	A*	B*	C*	D*
Residential density			34, 34	1	N/A	N/A	N/A
Land use mix diversity	34, 34, 62^M^, 62^F^, **62**^**F**^	**62**^**M**^, **62**^**F**^	**62**^**M**^	2	N/A	N/A	N/A
Street connectivity	**62**^**M**^, **62**^**F**^, **62**^**M**^, **62**^**F**^, 62^M^, 62^F^		34, 34	2	N/A	N/A	N/A
Access to shops/services/work	**58**, 58, 58, **62**^**M**^, **62**^**F**^, 62^M^, 62^F^, **88**^**2**^		34, 34, **57**, **88**^**1**^	5	8/12	67	+
Access to public transport			34, 34, 57	2	N/A	N/A	N/A
Access to recreation facilities			34, 34, 58, **62**^**M**^, **62**^**F**^	3	0/5	0	0
Walking/cycling facilities	57, **62**^**M**^, **62**^**F**^, 62^M^, 62^F^		34, 34, 34, 34, 58	4	5/10	50	(+)
Traffic-related safety	62^F^, **62**^**M**^, **62**^**F**^**, 62**^**M**^, **62**^**F**^**, 62**^**M**^, **62**^**F**^ ,	58, **62**^**M**^, **62**^**F**^, **62**^**M**^, **62**^**F**^, **62**^**M**^, **62**^**M**^	34, 34, 58, 62^M^, **62**^**F**^, **62**^**F**^	3	7/20	35	0
Crime-related safety	**62**^**F**^, 62^M^		34, 34, 58, **62**^**M**^, 62^F^, **88**^**1**^, **88**^**2**^	4	2/9	22	00
Aesthetics		**88**^**1**^	34, 34, 58, 62^M^, 62^F^**88**^**1**^, **88**^**1**^, **88**^**2**^, **88**^**2**^, **88**^**2**^	3	1/11	9	0
Urbanization	**62**^**M**^, **62**^**F**^	**51**^**M**^, **51**^**F**^	**62**^**M**^, **62**^**F**^	2	N/A	N/A	N/A

* A = n° of independent studies; B = n° of associated records divided by all records; C =% of evidence; D = summary code.

Regular vs italics font = subjective vs objective PA measures; regular vs bold font = subjective vs objective environmental measures.

^M^ = specific results for males; ^F^ = specific results for females; ^1^ = specific results for 1^st^ subgroup; ^2^ = specific results for 2^nd^ subgroup.

### Physical environment and the relationship with walking for transportation

Table 9 presents 11 studies that investigated the relationship between the physical environment and walking for transportation. Summary results demonstrated convincing evidence for a strong positive relationship with walkability. Further, transportation walking was unrelated to both access to shops/services/work and access to recreation facilities. Due to a lack of enough separate studies focusing on relationships between the other environmental factors and transportation walking, summary calculations were not applicable here.

**Table 9:** Summary results of evidence on the relationship environmental factors and transportation walking

**Environm. variables**	**Positive association**	**Negative association**	**No association**	A*	B*	C*	D*
Walkability	**78**, **82, 83**, 85, **86**		**82**	5	4/5	80	++
Street connectivity			85	1	N/A	N/A	N/A
Access to shops/services/work	63, 77	85	77	3	2/4	50	0
Access to public transport	50			1	N/A	N/A	N/A
Access to recreation facilities			**52**, **52**, 85, **92**, **92**, **92**, **92**, **92**, **92**, **92**,**92**, **92**, **92**	3	0/13	0	0
Walking/cycling facilities			85, 85	1	N/A	N/A	N/A
Safety			63, 85	2	N/A	N/A	N/A
Traffic-related safety			85	1	N/A	N/A	N/A
Crime-related safety			63, 85	2	N/A	N/A	N/A
Aesthetics	63	63, 85	63, 85	2	N/A	N/A	N/A
Urbanization	**84, 84**			1	N/A	N/A	N/A
Quality of environment			77^M^, 77^F^	1	N/A	N/A	N/A

* A = n° of independent studies; B = n° of associated records divided by all records; C = % of evidence; D = summary code.

Regular vs italics font = subjective vs objective PA measures; regular vs bold font = subjective vs objective environmental measures.

^M^ = specific results for males; ^F^ = specific results for females.

### Physical environment and the relationship with cycling for transportation

Summary results for the 19 studies assessing transportation cycling are depicted in Table 10. Convincing evidence for strong positive relationships of cycling for transportation were shown for walkability, access to shops/services/work and degree of urbanization. This latter finding means that people living in more urbanized areas tended to cycle more for transportation purposes. Possible evidence for a positive association was found between transportation cycling and walking/cycling facilities, whereas evidence showed a possible negative relationship for hilliness. There were no relationships with transportation cycling for access to public transport, access to recreation facilities, traffic- and crime-related safety and aesthetics. Too few individual studies examined relationships between the remaining factors and transportation cycling, so no summary results could be calculated there.

**Table 10:** Summary results of evidence on the relationship environmental factors and transportation cycling

**Environm. variables**	**Positive association**	**Negative association**	**No association**	A*	B*	C*	D*
Walkability	59, **83**, 85, **86**		**82**, **82**	5	4/6	67	++
Residential density	**53**^**M,1**^, **53**^**F,1**^		**53**^**M,2**^, **53**^**M,3**^, **53**^**F,2**^, **53**^**F,3**^	1	N/A	N/A	N/A
Land use mix diversity			80	1	N/A	N/A	N/A
Street connectivity	85			1	N/A	N/A	N/A
Access to shops/services/work	35, 35, 35, 38, **53**^**M,1**^, **53**^**F,1**^, **53**^**F,2**^, **53**^**F,3**^, ***64***, 77, 77, ***91***, ***91***, ***90***		**53**^**M,2**^, **53**^**M,3**^, 77, 77, 85	7	14/19	74	++
Access to public transport	50		35, **53**^**M,1**^, **53**^**M,2**^, **53**^**M,3**^, **53**^**F,1**^, **53**^**F,2**^, **53**^**F,3**^	3	1/8	13	0
Access to recreation facilities	85, ***91***	**52**	**52**, **92**, **92**, **92**, **92**, **92**, **92**, **92**, **92**, **92**, **92**	5	2/14	14	0
Walking/cycling facilities	***64***, ***64***, ***64***, 80, ***90***, ***91***		35, 35, ***64***, ***64***, ***64***, 80, 85, 85	6	6/14	43	(++)
Safety			80, 85	2	N/A	N/A	N/A
Traffic-related safety	**41**^**M**^, **41**^**F**^, ***64***, ***90***, ***91***	79^2^, ***91***, ***91***	35, 35, 35, 35, *64*, 79^1^, 80, 85	8	5/16	31	00
Crime-related safety	79^2^, 85, ***90***	***91***	35, 35, 79^1^, ***90***, ***91***	5	3/9	33	0
Aesthetics	79^1^	***91***	79^2^, 80	3	1/4	25	0
Urbanization	35, ***64***, **84**, ***90, 90, 91***, ***91***, ***91***		**84**	5	8/9	89	++
Hilliness	80	***64***, ***91***		3	2/3	67	(−)
Quality of environment			77^M^, 77^M^, 77^F^, 77^F^	1	N/A	N/A	N/A

* A = n° of independent studies; B = n° of associated records divided by all records; C = % of evidence; D = summary code.

Regular vs italics font = subjective vs objective PA measures; regular vs bold font = subjective vs objective environmental measures.

^M^ = specific results for males; ^F^ = specific results for females; ^1^ = specific results for 1^st^ subgroup; ^2^ = specific results for 2^nd^ subgroup; ^3^ = specific results for 3^rd^ subgroup.

## Discussion

During the past decade, researchers extensively studied the relationship between attributes of the physical environment and different domains of PA in developed countries. Various reviews have been published about this topic, but only a small proportion of findings in these publications refer to European studies. Reviews’ conclusions and recommendations for further research therefore mostly rely on North American and Australian study results. Given some clear differences in physical environmental design and physical activity behaviors between North America/Australia and Europe, this raises questions about the applicability of such results in a European context. To our knowledge, this is the first review that summarized specific European results on the relationships between attributes of the physical environment and PA. Despite this lack of European review evidence, the retrieval of 70 eligible papers for the present review shows that the research area is growing in this part of the world. Additionally, the fact that 60 out of the 70 studies were published after 2005 illustrates increasing interest in this topic during the last seven years and the need for an update on the literature in this continent-specific setting, which was the aim of the current review.

The discussion below provides a comparison between our Europe-specific results and outcomes of previous, non-continent-specific reviews, in order to reveal the most important differences and similarities. Our European summary results indicated convincing evidence for relationships with five environmental factors: *walkability* was positively related to total PA, transportation walking and transportation cycling, and *access to shops/services/work* was positively related to both general active transportation and transportation cycling. *Safety from traffic* showed a positive association with recreational walking/cycling, while evidence for *urbanization degree* revealed a positive relationship with transportation cycling and a negative relationship with total PA. Lastly, *quality of the environment* was positively related to total PA. This evidence primarily revealed that the majority of above-mentioned environmental attributes were more frequently associated with transportation PA, compared to recreational PA. For instance, the factors walkability, access to shops/services/work and urbanization degree were all unrelated to recreational PA, despite their associations with transportation PA. Most earlier reviews that focused mainly on non-European walking and cycling studies found comparable results. Associations of walkability with active travel were observed in the review of Panter and Jones [16] and two other non-Europe-specific reviews reported positive associations with utilitarian walking/biking trips, but not with trips for exercise or recreation [10,12]. A similar pattern in worldwide review evidence is present for access to shops/services/work. For instance, Duncan and colleagues [13] observed positive relationships with total PA, and reviews by Owen and colleagues [12] and Panter and Jones [16] identified positive associations with total walking and active travel, respectively. Saelens and Handy [15] found consistent associations with walking for transportation, whereas little or no evidence was observed with total and recreational walking. This patterning of findings for both non-Europe-specific as our European results might refer to a more prominent role of the above-mentioned environmental attributes in the transportation PA domain, rather than the recreational. Namely, higher walkability translates into a higher density and easier accessibility of destinations, such as work, which is especially inviting for PA with a transportation purpose. Recreational activities may be less dependent on the convenience of a route or the proximity of destinations. Moreover, it is possible that PA for recreational purposes is not necessarily undertaken in the neighborhood, whereas physical environmental measures mostly refer to the residential environment. In the same line, a higher degree of urbanization translates into denser areas, with more destinations that can be easily reached by bike, which might explain our convincing evidence on a positive relationship of urbanization degree with transportation cycling. However, our findings also indicated counter-intuitive evidence concerning urbanization, i.e., a negative association with total PA. Yet, in this case it is possible that occupational or domestic-oriented activities like gardening, rather than activities with a transportation or sports/exercise purpose, made the largest contribution to the total PA measures in the involved studies, and suburban or rural places lend themselves more for such pursuits than urban ones. Our convincing evidence on a positive relationship between quality of the environment and total PA contrasts with an earlier, non-Europe-specific review [9]. In that paper, the authors did not find a relationship between a similar environmental “combined scale” and total PA. Our results, however, might indicate that the cumulative contribution of several physical environmental aspects does have an impact on European health behavior, apart from the individual influence of separate environmental attributes. Yet, quality of the environment was insufficiently investigated in relation to other PA domains, which makes it hard to draw definite conclusions.

Our review’s summary results further yielded possible evidence on relationships with different PA domains for five environmental factors: *access to recreation facilities* was positively related to total PA, while *presence and quality of walking/cycling facilities* showed positive relationships with general active transportation and cycling for transportation. *Urbanization degree* was positively associated with total walking/cycling and the composite factor *quality of the environment* was positively related to LTPA. For the factor *hilliness*, possible evidence for a negative relationship was found with cycling for transportation. Similar to the convincing evidence, most possible relationships of environmental attributes were found in relation to transportation PA, rather than recreational PA. The positive relationship of active transport and transportation cycling with walking/cycling facilities and the negative association of transportation cycling with hilliness were found in the expected directions, as the results probably refer to the importance of respectively adequate infrastructure and the need for absence of difficulties during active transportation. Moreover, cycling for transport is a more common behavior in Europe compared to other continents [21], which may also explain the observed associations within the European studies. Worldwide evidence on the role of walking/cycling facilities is conflicting. One non-Europe-specific review [14] supports our findings by showing positive associations with commuting activities and a meta-analysis by Duncan and colleagues [13] observed positive relationships with total PA. By contrast, other reviews on worldwide studies identified either inconsistent results [16,103], or a lack of evidence for relationships with transportation PA, while positive relationships with recreational PA did appear [12,15]. Because of the above-mentioned inconsistencies in the existing worldwide review evidence and the fact that our own review results show less strong relationships (possible evidence), more studies need to reveal whether walking/cycling infrastructure plays an important role as a correlate of PA, and whether the strength of the relationship is more consistent for the transportation or recreational PA domain. In addition, more research on relationships between hilliness and PA is needed, as non-Europe-specific reviews also observed inconsistencies about the direction of associations with different PA domains [11,14].

Neighborhood *aesthetics* and *safety from crime* were the two environmental factors unrelated to several PA domains. In addition, the factors *access to recreation facilities* and *traffic safety* also showed a low importance in relation to specific PA domains. By way of comparison, concerning aesthetics, one non-Europe-specific review observed a lack of association between aesthetics and several PA domains [14] and another literature review [16] identified inconsistent relationships with active travel. Conversely, other worldwide literature reviews did find positive relationships with total PA [9,103] and with recreational and total walking, but not with transportation walking [12,15]. Furthermore, results in the non-Europe-specific literature are ambiguous when crime-related safety [9,15,104], as well as safety from traffic [9,12-16] are considered. Where certain reviews on worldwide studies found positive relationships between these two forms of safety and PA [9,13,104], others reported inconsistent or unrelated results regarding both safety from crime [15] and safety from traffic [12,14-16] with transportation and recreational PA. The present review’s unrelated results might indicate that, from a European PA perspective, aesthetics and safety levels are not so important. It is plausible that differences between low and high levels of these environmental factors are less pronounced in Europe, when compared to other geographical regions like North America, and other environmental attributes might outweigh the influence of these factors. Also the low importance of access to recreation facilities in the present review is in contrast with earlier non-Europe-specific reviews, where positive associations between access to recreation facilities and different PA domains did appear [9,13,14,16]. Especially the absence of relationships with recreational PA is unexpected. An explanation might again be that in Europe, leisure-time physical activity and recreational walking/cycling are done elsewhere than in the residential neighborhood, while most environmental measures refer to these residential areas, and environmental features of the places where recreational activities are undertaken may differ from those measured. Moreover, the recreational facilities may be situated too far from the home residence in order to be reached on foot or by bike, which could explain our unassociated findings in the domain of transportation PA. Another explanation for the European lack of associations is that the vast majority of these measurements were based on perceptions, so inter-individual differences in interpretations could contribute to the unrelated summary scores.

### Strengths

A first strength of the current study is its exclusive focus on European research, as it was not entirely clear yet if recommendations based on predominantly non-Europe-focused reviews would be applicable to the development of adequate interventions in this continent. Indeed, some of our summary results are conflicting with earlier, worldwide reviews, which supports the need for Europe-specific approaches. Secondly, we were able to summarize a large amount of studies that were previously unmentioned by other reviews in this research field, pointing out the importance of updating the evidence. Further, as researchers already have shown that particular domains of PA relate differently to certain measures of the physical environment [10,12,14], summarizing relationships according to specific domains of PA is a third strength of the current systematic review.

### Limitations

As some of the environmental factors were understudied in relation to specific PA domains, a first limitation of this systematic review is that we could not calculate a number of PA-domain-specific summary results. Therefore, we were not able to complete the existing worldwide evidence in all PA domains, regarding all included environmental attributes. A second limitation refers to the design of the studies: all included studies, except one, were cross-sectional. As a consequence, we could not ensure that our convincing and possible evidence refers to causal relationships between the environment and PA. Thirdly, as shown in Table 2, the largest part of studies were conducted in Western Europe (i.e., The U.K., Belgium and The Netherlands) while studies in Eastern Europe contributed the least to the total amount of publications. One of the reasons for this disproportion in geographical region might be the restriction for English-written publications. As a consequence, certain specific summary results (e.g., relationships between total PA and walkability) are dominantly determined by findings from Western European countries (e.g., Belgium). Since a broad inter-regional variety in cultural, policy and physical environmental aspects is present nowadays within the European continent itself, this overrepresentation may have biased the results. Therefore, caution should be paid when these results are generalized to other geographical areas, such as Eastern Europe. A last limitation of this review is that a quality assessment of the included studies was lacking. Therefore, some of the summary results may have been based upon the findings of methodological weaker studies, which, in turn, increased the risk of bias.

### Recommendations for future research

A first suggestion for future research is to expand the amount of European studies concerning relationships between physical environmental attributes and separate domains of PA. This review revealed that total PA was the most commonly used measure of PA, while some other domain-specific PA measures are lacking in relation to various environmental factors. Our findings show that following specific environmental attributes are still understudied in Europe: the three walkability components (i.e., residential density, land use mix diversity and street connectivity), access to public transportation, quality and presence of walking and cycling facilities, hilliness and general measures of the environmental quality. Future European research should therefore challenge the lack of studies on these attributes’ relations with domain-specific PA, in order to complete the existing worldwide evidence. Second, to encounter the above-mentioned underrepresentation of Eastern European studies, also the amount of studies on the relationship between the physical environment and different PA domains in this part of the continent should be expanded. A third recommendation relates to the used methods for assessment of environmental attributes. Our results indicated that the majority of included studies used environmental perceptions, while only a limited amount of studies included objective assessments of the physical environment. Objective and perceived measures of the physical environment have been shown to relate differently to PA [102], so an increase in studies combining both objective and subjective environmental measures is encouraged. Fourth, more longitudinal studies in the research field are needed in order to reveal the influence of (changes in) physical environmental attributes on different PA domains, in order to facilitate the development of appropriate and effective interventions for promoting PA. At last, given the fact that this review identified some clear inter-continental differences concerning the relationship between the physical environment and domain-specific PA in adults, it is plausible that the environmental correlates of PA in other age groups might also be continent- or region-specific. Hence, it would be interesting to compare the current review evidence on adults with that of other age groups, such as young adults with little children, or older adults.

## Conclusions

Our summary results revealed several relationships between the physical environment and different domains of PA. Transportation PA, rather than PA with a recreational purpose, appears to be more consistently related to the physical environment. First, convincing evidence on positive relationships between particular domains of PA emerged for the environmental attributes walkability, access to shops/services/work and environmental quality, and this evidence contributed to a more complete view on the existing evidence worldwide. However, causal relationships could not be revealed yet, because longitudinal studies were absent. Next, there was possible evidence on a positive relationship for transportation PA and walking/cycling facilities, and on a negative relationship of transportation cycling with hilliness. Although these findings seem promising in completing existing knowledge, this possible evidence is less strong than the convincing results we found for other environmental factors, such as walkability, and needs more rigorous investigation before generalizations can be made. The lack of associations between domain-specific PA and access to recreation facilities, aesthetics, crime- and traffic-related safety was contrasting with earlier, non-Europe-specific reviews. This suggests that these factors might play a less important role from a European PA perspective. At last, the relationship between a considerable amount of environmental attributes and particular PA domains is still understudied in European research. Therefore, increasing research on relationships between PA and the three walkability components (i.e., residential density, land use mix diversity and street connectivity), access to public transportation, quality and presence of walking and cycling facilities, hilliness and general measures of the environmental quality is highly encouraged.

## Abbreviations

PA: Physical activity; MVPA: Moderate-to-vigorous physical activity; LTPA: Leisure-time physical activity.

## Competing interests

The authors declare that they have no competing interests.

## Authors’ contributions

IDB, BD, JVC and VVH developed the search strategy; VVH and JVC conducted the systematic search of electronic databases and screening; VVH undertook the data extraction, summary calculation, drafting of the tables and writing of the manuscript, supervised by IDB and BD; IDB, BD, JVC, LG, LM and NVdW contributed to critical revision of the manuscript, and all approved the final version.

## Pre-publication history

The pre-publication history for this paper can be accessed here:

http://www.biomedcentral.com/1471-2458/12/807/prepub

## Supplementary Material

Additional file 1 **Detailed characteristics of the original studies in the systematic review.** Description: This table contains more detailed information about following study characteristics: sample size, mean age participants, % females, sampling methods, country, design, dependent and independent variables and results (associations). (PDF 1016 kb) Click here for file

## References

[B1] U.S.Department Of Health And Human Services Physical Activity and Health: A Report of the Surgeon General 1996 Atlanta, Georgia: USDHSS, Centers for Disease Control and Prevention

[B2] Warburton D Nicol C Bredin S Health benefits of physical activity: the evidence Can Med Assoc J 2006 174 801 809 10.1503/cmaj.051351 16534088 PMC1402378

[B3] Oja P Titze S Bauman A de Geus B Krenn P Reger-Nash B Health benefits of cycling: a systematic review Scand J Med Sci Spor 2011 21 496 509 10.1111/j.1600-0838.2011.01299.x 21496106

[B4] Physical Inactivity: a global public health problem http://www.who.int/dietphysicalactivity/factsheet_inactivity/en/index.html

[B5] McLeroy K Bibeau D Steckler A Glanz K An ecological perspective on health promotion programs Health Educ Q 1988 15 351 377 10.1177/109019818801500401 3068205

[B6] Sallis JF Cervero RB Ascher W Henderson KA Kraft MK Kerr J An ecological approach to creating active living communities Annu Rev Public Health 2006 27 297 322 10.1146/annurev.publhealth.27.021405.102100 16533119

[B7] Richard L Gauvin L Raine K Ecological models revisited: their uses and evolution in health promotion over two decades Annu Rev Public Health 2011 307 326 21219155 10.1146/annurev-publhealth-031210-101141

[B8] Davison KK Lawson CT Do attributes in the physical environment influence children's physical activity? A review of the literature Int J Behav Nutr Phys Act 2006 3 19 10.1186/1479-5868-3-19 16872543 PMC1557665

[B9] Humpel N Owen N Leslie E Environmental factors associated with adults' participation in physical activity - A review Am J Prev Med 2002 22 188 199 10.1016/S0749-3797(01)00426-3 11897464

[B10] Saelens BE Sallis JF Frank LD Environmental correlates of walking and cycling: Findings from the transportation, urban design, and planning literatures Ann Behavioral Med 2003 25 80 91 10.1207/S15324796ABM2502_03 12704009

[B11] McCormack G Giles-Corti B Lange A Smith T Martin K Pikora TJ An update of recent evidence of the relationship between objective and self-report measures of the physical environment and physical activity behaviours J Sci Med Sport 2004 7 81 92 15214606 10.1016/s1440-2440(04)80282-2

[B12] Owen N Humpel N Leslie E Bauman A Sallis JF Understanding environmental influences on walking - Review and research agenda Am J Prev Med 2004 27 67 76 10.1016/j.amepre.2004.03.006 15212778

[B13] Duncan MJ Spence JC Mummery WK Perceived environment and physical activity: a meta-analysis of selected environmental characteristics Int J Behav Nutr Phys Act 2005 2 11 10.1186/1479-5868-2-11 16138933 PMC1236952

[B14] Wendel-Vos W Droomers M Kremers S Brug J van Lenthe F Potential environmental determinants of physical activity in adults: a systematic review Obes Rev 2007 8 425 440 10.1111/j.1467-789X.2007.00370.x 17716300

[B15] Saelens BE Handy SL Built environment correlates of walking: A review Med Sci Sports Exerc 2008 40 S550 S566 10.1249/MSS.0b013e31817c67a4 18562973 PMC2921187

[B16] Panter JR Jones A Attitudes and the environment as determinants of active travel in adults: what do and don't we know? J Phys Act Health 2010 7 551 561 20683098 10.1123/jpah.7.4.551

[B17] Cummins S Macintyre S Food environments and obesity - neighbourhood or nation? Int J Epidemiol 2006 35 100 104 16338945 10.1093/ije/dyi276

[B18] Rodrigue J-P Comtois C Slack B The Geography of Transport Systems 2009 2 New York: Routledge

[B19] Pucher J Buehler R Making cycling irresistible: Lessons from the Netherlands, Denmark and Germany Transp Rev 2008 28 495 528 10.1080/01441640701806612

[B20] Forsyth A Krizek K Promoting walking and bicyling: assessing the evidence to assist planners Built Environment 2010 36 429 446 10.2148/benv.36.4.429

[B21] Bassett DR Pucher J Buehler R Thompson DL Crouter SE Walking, cycling, and obesity rates in Europe, North America, and Australia J Phys Act Health 2008 5 795 814 19164816 10.1123/jpah.5.6.795

[B22] Sugiyama T Neuhaus M Cole R Giles-Corti B Owen N Destination and Route Attributes Associated with Adults' Walking: A Review Med Sci Sports Exer 2012 44 1275 1286 10.1249/MSS.0b013e318247d286 22217568

[B23] Moher D Liberati A Tetzlaff J Altman DG Preferred Reporting Items for Systematic Reviews and Meta-Analyses: The PRISMA Statement J Clin Epid 2009 62 1006 1012 10.1016/j.jclinepi.2009.06.005 19631508

[B24] Bamana A Tessier S Vuillemin A Association of perceived environment with meeting public health recommendations for physical activity in seven European countries J Public Health 2008 30 274 281 10.1093/pubmed/fdn041 18544614

[B25] Bergman P Grjibovski AM Hagstromer M Bauman A Sjostrom M Adherence to physical activity recommendations and the influence of socio-demographic correlates - a population-based cross-sectional study BMC Public Health 2008 8 367 10.1186/1471-2458-8-367 18945354 PMC2576236

[B26] Bergman P Grjibovski AM Hagstromer M Sallis JF Sjostrom M The association between health enhancing physical activity and neighbourhood environment among Swedish adults - a population-based cross-sectional study Int J Behav Nutr Phys Act 2009 6 8 10.1186/1479-5868-6-8 19203354 PMC2647520

[B27] Bertrais S Preziosi P Mennen L Galan P Hercberg S Oppert JM Sociodemographic and geographic correlates of meeting current recommendations for physical activity in middle-aged French adults: the Supplementation en Vitamines et Mineraux Antioxydants (SUVIMAX) Study Am J Public Health 2004 94 1560 1566 10.2105/AJPH.94.9.1560 15333315 PMC1448494

[B28] Bjork J Albin M Grahn P Jacobsson H Ardo J Wadbro J Recreational values of the natural environment in relation to neighbourhood satisfaction, physical activity, obesity and wellbeing J Epidemiol Community Health 2008 62 e2 10.1136/jech.2007.062414 18365329

[B29] Bolivar J Daponte A Rodriguez M Sanchez JJ The influence of individual, social and physical environment factors on physical activity in the adult population in Andalusia, Spain Int J Environ Res Public Health 2010 7 60 77 10.3390/ijerph7010060 20195433 PMC2819776

[B30] Bonnefoy XR Braubach M Moissonnier B Monollbaev K Robbel N Housing and health in Europe: preliminary results of a pan-European study Am J Public Health 2003 93 1559 1563 10.2105/AJPH.93.9.1559 12948980 PMC1448010

[B31] Cochrane T Davey RC Gidlow C Smith GR Fairburn J Armitage CJ Small area and individual level predictors of physical activity in urban communities: a multi-level study in Stoke on Trent, England Int J Environ Res Public Health 2009 6 654 677 10.3390/ijerph6020654 19440408 PMC2672366

[B32] Coombes E Jones AP Hillsdon M The relationship of physical activity and overweight to objectively measured green space accessibility and use Soc Sci Med 2010 70 816 822 10.1016/j.socscimed.2009.11.020 20060635 PMC3759315

[B33] De Bourdeaudhuij I Sallis JF Saelens BE Environmental correlates of physical activity in a sample of Belgian adults Am J Health Promot 2003 18 83 92 10.4278/0890-1171-18.1.83 13677966

[B34] De Bourdeaudhuij I Teixeira PJ Cardon G Deforche B Environmental and psychosocial correlates of physical activity in Portuguese and Belgian adults Public Health Nutr 2005 8 886 895 16277805 10.1079/phn2005735

[B35] de Geus B De Bourdeaudhuij I Jannes C Meeusen R Psychosocial and environmental factors associated with cycling for transport among a working population Health Educ Res 2008 23 697 708 17947248 10.1093/her/cym055

[B36] Dygryn J Mitas J Stelzer J The influence of built environment on walkability using Geographic Information System J Hum Kinet 2010 24 93 99 10.2478/v10078-010-0025-2

[B37] Ellaway A Macintyre S Bonnefoy X Graffiti, greenery, and obesity in adults: secondary analysis of European cross sectional survey Br Med J 2005 331 611 612 10.1136/bmj.38575.664549.F7 16113034 PMC1215553

[B38] Engbers LH Hendriksen IJ Characteristics of a population of commuter cyclists in the Netherlands: perceived barriers and facilitators in the personal, social and physical environment Int J Behav Nutr Phys Act 2010 7 89 10.1186/1479-5868-7-89 21143948 PMC3012015

[B39] Foster C Hillsdon M Thorogood M Environmental perceptions and walking in English adults J Epidemiol Community Health 2004 58 924 928 10.1136/jech.2003.014068 15483308 PMC1732623

[B40] Foster C Hillsdon M Jones A Grundy C Wilkinson P White M Objective measures of the environment and physical activity - results of the Environment and Physical Activity Study in English adults J Phys Act Health 2009 6 S70 S80 19998852 10.1123/jpah.6.s1.s70

[B41] Foster CE Panter JR Wareham NJ Assessing the impact of road traffic on cycling for leisure and cycling to work Int J Behav Nutr Phys Act 2011 8 61 10.1186/1479-5868-8-61 21663654 PMC3127970

[B42] Fromel K Mitas J Kerr J The associations between active lifestyle, the size of a community and SES of the adult population in the Czech Republic Health Place 2009 15 447 454 10.1016/j.healthplace.2008.08.003 18842449

[B43] Gast GC Frenken FJ van Leest LA Wendel-Vos GC Bemelmans WJ Intra-national variation in trends in overweight and leisure time physical activities in The Netherlands since 1980: stratification according to sex, age and urbanisation degree Int J Obes (Lond) 2007 31 515 520 10.1038/sj.ijo.0803429 16819527

[B44] Gidlof-Gunnarsson A Ohrstrom E Noise and well-being in urban residential environments: the potential role of perceived availability to nearby green areas Landsc Urban Plan 2007 83 115 126 10.1016/j.landurbplan.2007.03.003

[B45] Guthold R Ono T Strong KL Chatterji S Morabia A Worldwide variability in physical inactivity - A 51-country survey Am J Prev Med 2008 34 486 494 10.1016/j.amepre.2008.02.013 18471584

[B46] Harrison RA Gemmell I Heller RF The population effect of crime and neighbourhood on physical activity: an analysis of 15,461 adults J Epidemiol Community Health 2007 61 34 39 10.1136/jech.2006.048389 17183012 PMC2465585

[B47] Hillsdon M Panter J Foster C Jones A The relationship between access and quality of urban green space with population physical activity Public Health 2006 120 1127 1132 10.1016/j.puhe.2006.10.007 17067646

[B48] Jones A Hillsdon M Coombes E Greenspace access, use, and physical activity: Understanding the effects of area deprivation Prev 2009 49 500 505 10.1016/j.ypmed.2009.10.012 PMC3748371 19857513

[B49] Kamphuis CB van Lenthe FJ Giskes K Huisman M Brug J Mackenbach JP Socioeconomic status, environmental and individual factors, and sports participation Med Sci Sports Exerc 2008 40 71 81 18182936 10.1249/mss.0b013e318158e467

[B50] Keijer MJN Rietveld P How do people get to the railway station? The Dutch experience Transport Plan Techn 2000 23 215 235 10.1080/03081060008717650

[B51] Kwasniewska M Kaczmarczyk-Chalas K Pikala M Broda G Kozakiewicz K Pajak A Socio-demographic and lifestyle correlates of commuting activity in Poland Prev Med 2010 50 257 261 10.1016/j.ypmed.2010.02.011 20219528

[B52] Maas J Verheij RA Spreeuwenberg P Groenewegen PP Physical activity as a possible mechanism behind the relationship between green space and health: A multilevel analysis BMC Public Health 2008 8 206 10.1186/1471-2458-8-206 18544169 PMC2438348

[B53] Maat K Timmermans HJP Influence of the residential and work environment on car use in dual-earner households Transp Res: Part A: Pol Practice 2009 43 654 664 10.1016/j.tra.2009.06.003

[B54] Mason P Kearns A Bond L Neighbourhood walking and regeneration in deprived communities Health Place 2011 17 727 737 10.1016/j.healthplace.2011.01.010 21398165

[B55] Miles R Neighborhood disorder, perceived safety, and readiness to encourage use of local playgrounds Am J Prev Med 2008 34 275 281 10.1016/j.amepre.2008.01.007 18374240

[B56] Milosevic M Golubic R Mustajbegovic J Jelinic JD Holcer NJ Kern J Regional pattern of physical inactivity in Croatia Coll Antropol 2009 33 35 38 19563144

[B57] Molina-Garcia J Castillo I Sallis JF Psychosocial and environmental correlates of active commuting for university students Prev Med 2010 51 136 138 10.1016/j.ypmed.2010.05.009 20510271

[B58] Ogilvie D Mitchell R Mutrie N Petticrew M Platt S Personal and environmental correlates of active travel and physical activity in a deprived urban population Int J Behav Nutr Phys Act 2008 5 43 10.1186/1479-5868-5-43 18752663 PMC2538543

[B59] Owen N De Bourdeaudhuij I Sugiyama T Leslie E Cerin E Van Dyck D Bauman A Bicycle use for transport in an Australian and a Belgian city: associations with built-environment attributes J Urban Health 2010 87 189 198 10.1007/s11524-009-9424-x 20174879 PMC2845830

[B60] Panter J Jones A Hillsdon M Equity of access to physical activity facilities in an English city Prev Med 2008 46 303 307 10.1016/j.ypmed.2007.11.005 18096216

[B61] Panter JR Jones AP Associations between physical activity, perceptions of the neighbourhood environment and access to facilities in an English city Soc Sci Med 2008 67 1917 1923 10.1016/j.socscimed.2008.09.001 18835074

[B62] Panter JR Jones AP Van Sluijs EM Griffin SJ Wareham NJ Environmental and psychological correlates of older adult's active commuting Med Sci Sports Exerc 2011 43 1235 1243 10.1249/MSS.0b013e3182078532 21131863 PMC3842528

[B63] Parkes A Kearns A The multi-dimensional neighbourhood and health: a cross-sectional analysis of the Scottish Household Survey, 2001 Health Place 2006 12 1 18 10.1016/j.healthplace.2004.03.004 16243677

[B64] Parkin J Wardman M Page M Estimation of the determinants of bicycle mode share for the journey to work using census data Transportation 2008 35 93 109

[B65] Pascual C Regidor E Martinez D Elisa CM Dominguez V Socioeconomic environment, availability of sports facilities, and jogging, swimming and gym use Health Place 2009 15 553 561 10.1016/j.healthplace.2008.08.007 18986825

[B66] Pitsavos C Panagiotakos DB Lentzas Y Stefanadis C Epidemiology of leisure-time physical activity in socio-demographic, lifestyle and psychological characteristics of men and women in Greece: the ATTICA Study BMC Public Health 2005 5 37 10.1186/1471-2458-5-37 15836794 PMC1087851

[B67] Poortinga W Perceptions of the environment, physical activity, and obesity Soc Sci Med 2006 63 2835 2846 10.1016/j.socscimed.2006.07.018 16952415

[B68] Rutten A Abel T Kannas L von Lengerke T Luschen G Diaz JAR Self reported physical activity, public health, and perceived environment: results from a comparative European study J Epidemiol Community Health 2001 55 139 146 10.1136/jech.55.2.139 11154254 PMC1731821

[B69] Rutten A Abu-Omar K Perceptions of environmental opportunities for physical activity in the European Union Soz Praventiv Med 2004 49 310 317 10.1007/s00038-004-3101-3 15497650

[B70] Santana P Santos R Nogueira H The link between local environment and obesity: a multilevel analysis in the Lisbon Metropolitan Area, Portugal Soc Sci Med 2009 68 601 609 10.1016/j.socscimed.2008.11.033 19135287

[B71] Santos MS Vale MS Miranda L Mota J Socio-demographic and perceived environmental correlates of walking in Portuguese adults–a multilevel analysis Health Place 2009 15 1094 1099 10.1016/j.healthplace.2009.05.009 19540147

[B72] Santos R Silva P Santos P Ribeiro JC Mota J Physical activity and perceived environmental attributes in a sample of Portuguese adults: Results from the Azorean Physical Activity and Health Study Prev Med 2008 47 83 88 10.1016/j.ypmed.2008.02.027 18400284

[B73] Shenassa ED Liebhaber A Ezeamama A Perceived safety of area of residence and exercise: a pan-European study Am J Epidemiol 2006 163 1012 1017 10.1093/aje/kwj142 16571742

[B74] Sigmundova D El Ansari W Sigmund E Neighbourhood environment correlates of physical activity: a study of eight Czech regional towns Int J Environ Res Public Health 2011 8 341 357 10.3390/ijerph8020341 21556190 PMC3084465

[B75] Stafford M Cummins S Ellaway A Sacker A Wiggins RD Macintyre S Pathways to obesity: Identifying local, modifiable determinants of physical activity and diet Soc Sci Med 2007 65 1882 1897 10.1016/j.socscimed.2007.05.042 17640787

[B76] Stahl T Rutten A Nutbeam D Bauman A Kannas L Abel T The importance of the social environment for physically active lifestyle - results from an international study Soc Sci Med 2001 52 1 10 10.1016/S0277-9536(00)00116-7 11144909

[B77] Stronegger WJ Titze S Oja P Perceived characteristics of the neighborhood and its association with physical activity behavior and self-rated health Health Place 2010 16 736 743 10.1016/j.healthplace.2010.03.005 20378392

[B78] Sundquist K Eriksson U Kawakami N Skog L Ohlsson H Arvidsson D Neighborhood walkability, physical activity, and walking behavior: the Swedish Neighborhood and Physical Activity (SNAP) study Soc Sci Med 2011 72 1266 1273 10.1016/j.socscimed.2011.03.004 21470735

[B79] Titze S Stronegger WJ Janschitz S Oja P Environmental, social, and personal correlates of cycling for transportation in a student population J Phys Act Health 2007 4 66 79 17489008 10.1123/jpah.4.1.66

[B80] Titze S Stronegger WJ Janschitz S Oja P Association of built-environment, social-environment and personal factors with bicycling as a mode of transportation among Austrian city dwellers Prev Med 2008 47 252 259 10.1016/j.ypmed.2008.02.019 18417199

[B81] Toftager M Ekholm O Schipperijn J Stigsdotter U Bentsen P Gronbaek M Distance to green space and physical activity: a danish national representative survey J Phys Act Health 2011 8 741 749 21832288 10.1123/jpah.8.6.741

[B82] Van Dyck D Deforche B Cardon G De Bourdeaudhuij I Neighbourhood walkability and its particular importance for adults with a preference for passive transport Health Place 2009 15 496 504 10.1016/j.healthplace.2008.08.010 18974020

[B83] Van Dyck D Cardon G Deforche B Sallis JF Owen N De Bourdeaudhuij I Neighborhood SES and walkability are related to physical activity behavior in Belgian adults Prev Med 2010 50 Suppl 1 S74 S79 19751757 10.1016/j.ypmed.2009.07.027

[B84] Van Dyck D Cardon G Deforche B De Bourdeaudhuij I Urban–rural differences in physical activity in Belgian adults and the importance of psychosocial factors J Urban Health 2011 88 154 167 10.1007/s11524-010-9536-3 21274644 PMC3042086

[B85] Van Dyck D Cardon G Deforche B Giles-Corti B Sallis JF Owen N Environmental and psychosocial correlates of accelerometer-assessed and self-reported physical activity in Belgian adults Int J Behav Med 2011 8 235 245 21038103 10.1007/s12529-010-9127-4

[B86] Van Dyck D Cardon G Deforche B Owen N De Bourdeaudhuij I Relationships between neighborhood walkability and adults' physical activity: How important is residential self-selection? Health Place 2011 17 1011 1014 10.1016/j.healthplace.2011.05.005 21596613

[B87] Van Dyck D Cardon G Deforche B De Bourdeaudhuij I Do adults like living in high-walkable neighborhoods? Associations of walkability parameters with neighborhood satisfaction and possible mediators Health Place 2011 17 971 977 10.1016/j.healthplace.2011.04.001 21570333

[B88] van Lenthe FJ Brug J Mackenbach JP Neighbourhood inequalities in physical inactivity: the role of neighbourhood attractiveness, proximity to local facilities and safety in the Netherlands Soc Sci Med 2005 60 763 775 10.1016/j.socscimed.2004.06.013 15571894

[B89] Van Tuyckom C Macro-environmental factors associated with leisure-time physical activity: a cross-national analysis of EU countries Scand J Public Health 2011 39 419 426 10.1177/1403494810396553 21273227

[B90] Vandenbulcke G Thomas I de Geus B Degraeuwe B Torfs R Meeusen R Mapping bicycle use and the risk of accidents for commuters who cycle to work in Belgium Transp Pol 2009 16 77 87 10.1016/j.tranpol.2009.03.004

[B91] Vandenbulcke G Cycle commuting in Belgium: spatial determinants and "re-cycling" strategies Transp Res: Part A: Pol Practice 2011 45 118 137 10.1016/j.tra.2010.11.004

[B92] Wendel-Vos GC Schuit AJ de Niet R Boshuizen HC Saris WH Kromhout D Factors of the physical environment associated with walking and bicycling Med Sci Sports Exerc 2004 36 725 730 10.1249/01.MSS.0000121955.03461.0A 15064601

[B93] Wendel-Vos GCW van Hooijdonk C Uitenbroek D Agyemang C Lindeman EM Droomers M Environmental attributes related to walking and bicycling at the individual and contextual level J Epidemiol Community Health 2008 62 689 694 10.1136/jech.2007.062869 18621953

[B94] Saelens BE Sallis JF Black JB Chen D Neighborhood-based differences in physical activity: An environment scale evaluation Am J Public Health 2003 93 1552 1558 10.2105/AJPH.93.9.1552 12948979 PMC1448009

[B95] Cerin E Saelens BE Sallis JF Frank LD Neighborhood environment walkability scale: Validity and development of a short form Med Sci Sports Exerc 2006 38 1682 1691 10.1249/01.mss.0000227639.83607.4d 16960531

[B96] Cerin E Conway TL Saelens BE Frank LD Sallis JF Cross-validation of the factorial structure of the Neighborhood Environment Walkability Scale (NEWS) and its abbreviated form (NEWS-A) Int J Behav Nutr Phys Act 2009 6 32 10.1186/1479-5868-6-32 19508724 PMC2700069

[B97] de Melo LL Menec V Porter MM Ready AE Personal factors, perceived environment, and objectively measured walking in old age J Aging Phys Act 2010 18 280 291 20651415 10.1123/japa.18.3.280

[B98] Cerin E Sit CH Cheung MC Ho SY Lee LC Chan WM Reliable and valid NEWS for Chinese seniors: measuring perceived neighborhood attributes related to walking Int J Behav Nutr Phys Act 2010 7 84 10.1186/1479-5868-7-84 21108800 PMC2999582

[B99] Reis JP Bowles HR Ainsworth BE Dubose KD Smith S Laditka JN Nonoccupational physical activity by degree of urbanization and U.S. geographic region Med Sci Sports Exerc 2004 12 2093 2098 15570145 10.1249/01.mss.0000147589.98744.85

[B100] Pont K Ziviani J Wadley D Bennett S Abbott R Environmental correlates of children's active transportation: A systematic literature review Health Place 2009 15 849 862 10.1016/j.healthplace.2009.02.002 19285904

[B101] Prins RG Oenema A van der Horst K Brug J Objective and perceived availability of physical activity opportunities: differences in associations with physical activity behavior among urban adolescents Int J Behav Nutr Phys Act 2009 6 70 10.1186/1479-5868-6-70 19832969 PMC2770555

[B102] Gebel K Bauman A Owen N Correlates of non-noncordance between perceived and objective measures of walkability Ann Behav Med 2009 37 228 238 10.1007/s12160-009-9098-3 19396503

[B103] McCormack GR Shiell A In search of causality: a systematic review of the relationship between the built environment and physical activity among adults Int J Behav Nutr Phys Act 2011 8 125 10.1186/1479-5868-8-125 22077952 PMC3306205

[B104] Loukaitou-Sideris A Eck JE Crime prevention and active living Am J Health Promot 2007 21 380 389 10.4278/0890-1171-21.4s.380 17465184

