# Supplementary material for: Relationship between the physical environment and different domains of physical activity in European adults: a systematic review
Source: BMC Public Health. 2012 Sep 19;12:807. doi: 10.1186/1471-2458-12-807 (PMC3507898; doi:10.1186/1471-2458-12-807)
Supplement: Additional file 1 — Detailed characteristics of the original studies in the systematic review. Description: This table contains more detailed information about following study characteristics: sample size, mean age participants, % females, sampling methods, country, design, dependent and independent variables and results (associations). (PDF 1016 kb) [file 1471-2458-12-807-S1.pdf]

Study	Participants	Sampling method	Setting/Country	Study design	Dependent variables	Independent variables	Relationship (+, 0, -)	Comments
**Bamana et al. 2008**<br><br>**Ref n° 24**	- n = 4,231<br>- *M* age= 44.7 ± 16.9y<br>- 56.5% F	- random (random digit dialling)<br><br>- r.r. not provided	Finland (n= 584), France (n=550); Germany (n=628); Italy (n=593); Netherlands (n=611); Spain (n=598); England (n=667)<br><br>Data collection June- Nov 2000	CS	*Subjective:*<br>**TOTAL PA**<br>(short IPAQ, telephone version last 7d)<br><br>- meeting PHRs*[1]	*Subjective:*<br>**ACCESS RECR FACIL**<br>- "area where I live offers many opportunities to be physically active"	<u>*Bivariate binary logistic regression analyses*</u><br>*(adj for country and month of survey)*<br>- area where I live offers many opportunities to be PA **(+)**	*[1] meeting PHRs = ≥3 d/wk of vig PA accumulating ≥ 1500 METmin/wk; OR 7d/wk of any combination of walking, mod or vig intensity activities achieving ≥3000METmin/wk<br>- Cronbach's Alpha for the physical and policy environment scale = 0.74<br>- all environmental variables were subdivided into tertiles, of which the lowest ("not true at all") = the reference category
**Bergman et al. 2008**<br><br>**Ref n° 25**	- n= 1,470<br>- *M* age= 46± 15y<br>- 52.9% F	- random<br>- r.r.= 59%	Sweden; IPS (International PA Prevalence Study)<br><br>Data collection Oct – Nov 2003	CS	*Subjective:*<br>**TOTAL PA**<br>(short IPAQ, self-administered version)<br><br>- being moderately PA*[1]<br>- being highly PA*[2]	*Subjective:*<br>**URBANIZATION**<br>- residential community size*[3]	<u>*Multinomial logistic regression*</u><br>*(crude analyses)*<br><u>Moderate PA:</u><br>- residential community size (0)<br><u>High PA</u>:<br>- residential community size **(-)**	*[1] <u>moderate PA</u>: ≥3 d/wk of vig PA for ≥ 20min/d; OR ≥5d/wk of mod intensity PA or walking for ≥ 30min/d; OR ≥5d/wk of any combination of walking, mod or vig intensity activities achieving ≥ 600METmin/wk<br>*[2] <u>high PA</u>: ≥3 d/wk of vig PA accumulating ≥ 1500 METmin/wk; OR 7d/wk of any combination of walking, mod or vig intensity activities achieving ≥3000METmin/wk<br>*[3] residential community size was categorized as [1]"large town": > 100,000 inhabitants; [2] "medium-sized town": 30,000 – 100,000 inhab.; [3] "small-sized town": 1,000 – 30,000 inhab.; [4] "village": < 1,000 inhab.<br>- "large town" = reference category
**Bergman et al. 2009**<br><br>**Ref n°26**	- n= 1,470<br>- *M* age= 46± 15y<br>- 52.9% F	- random<br>- r.r.= 59%	Sweden; IPS (International PA Prevalence Study)<br><br>Data collection	CS	*Subjective:*<br>(short IPAQ, self-administered version)<br>**TOTAL WALKING:**<br>- walking*[1]	*Subjective*[3]:*<br>**URBANIZATION**<br>- urbanization degree local area<br>**TRAFFIC SAFETY**	<u>*Multinomial logistic regression*</u><br>*(Crude ORs)*<br><u>Walking:</u><br>- urbanization **(+)**<br>- traffic safety (0)	*[1] low walking < 80 min/wk; moderate walking 80-300 min/wk; high walking > 300min/wk (=upper tertile)<br>*[2] recommendation for being

			Oct – Nov 2003		**TOTAL PA:**<br>- achieving HEPA norm: mod PA *[2]	- traffic intensity local area<br>**WALKING/CYCLING FACIL**<br>- opportunities and aesthetics local area<br>**CRIME SAFETY**<br>- fear of crime local area	- opportunities & aesthetics **(+)**<br>- safety from crime (0)<br><br><u>HEPA:</u><br>- urbanization **(-)**<br>- traffic safety **(+)**<br>- opportunities & aesthetics (0)<br>- safety from crime **(+)**	moderately physically active: ≥3 d/wk of vig PA for ≥ 20min/d; OR ≥5d/wk of mod intensity PA or walking for ≥ 30min/d; OR ≥5d/wk of any combination of walking, mod or vig intensity activities achieving ≥ 600METmin/wk<br>*[3]”local neighborhood” = area within a 15-min walk from home<br>- upper tertile of the variables = reference category<br>()= specific results lower tertile of walking (HEPA)<br>- only upper and lower tertiles are taken up
**Bertrais et al. 2004**<br><br>**Ref n° 27**	- n_{total}l = 7,404<br>- age range = 45y – 68y<br>- *M* age Men: 55.4y ± 4.7<br>Women: 53.2y ± 5.3<br>- 54.0% F	-random<br>- r.r. not provided	France<br>SUVIMAX study<br><br>Data collection 1998	CS	*Subjective:*<br>**LTPA**<br>(self-administered MAQ)<br>- meeting public health recommendations*[1]<br>(= mod or vig **LTPA**)	*Objective:*<br>**URBANIZATION**<br>- urbanization degree*[2]	<u>*Logistic regression analyses*</u><br>*(crude ORs)*<br><br>*MEN*:<br>- urbanization (0)<br><br>*WOMEN*:<br>- urbanization **(-)**	- MAQ = Modifiable Activity Questionnaire (Kriska et al., 1990)<br>*[1] *inactivity* [no LTPA]; *irregular activity* [some LTPA but below “moderate activity”]; *moderate activity* [≥ 150 min/wk of LTPA > 3 METs but below “vigorous activity”]; *vigorous activity* [≥ 60min/wk of LTPA > 6METs during ≥ 20 min per session]<br>*[2] <u>urbanization</u>: *urban poles* [urban units – 1 or more municipalities – that offer at least 5000 jobs]; *periurban zones* [municipalities surrounding urban pole]; *multipolarized areas* [municipalities located outside urban unit, in which at least 40% of the res. population works in an urban area]; *rural municipalities* [all other]<br>- for the analyses, “urban pole”= reference category
**Björk et al. 2008**<br><br>**Ref n° 28**	-n = 24,819<br>- *M* age= 49y ± 16.6<br>- 54.3% F	- Clusters: purposeful<br>- Individuals: Random	**Suburban and rural** areas of Scania, southern Sweden	CS	*Subjective*<br>**TOTAL PA**<br>- time spent on moderate PA/wk *[1]	*Objective*<br>**ACCESS RECR FACIL**<br>(GIS)<br>- n° of recreational values	<u>*Spearman’s rank correlations*</u><br>- n° recreational values present within 300m distance **(+)**<br>- n° recreational values present	*[1] question about MPA/wk: “On an ordinary week, how much time do you spend on moderately demanding PA’s? (e.g. walking quickly,

		- r.r. 59% (27,963)	Data collection Sept 2004 – Jan 2005			present near the residence (100-300m from centre of property)*[2]	within 100m distance **(+)**	gardening, heavier household work, cycling, swimming)<br>*[2]Using GIS, definitions of five recreational values of the close natural environment within 100-300 meters of the home residence were established: [1]serene, [2]wild, [3]lush, [4]spacious and [5]culture
**Bolívar et al. 2010**<br><br>**Ref n° 29**	-n= 13,193<br>- Men:<br>n = 6425<br>M age= 42.84y ± 18.37<br><br>- Women<br>n = 6768<br>- M age= 45.03y ± 19.14<br><br>- 51.3% F	- probabilistic, stratified and multi-stage<br><br>- r.r. = not provided	Andalusia Health Survey, Spain<br><br>Data collection 1999 and 2003	CS	*Subjective:*<br>**LTPA**<br>- PA during *free time* *[1]	*Objective:*<br>**URBANIZATION**<br>- size of municipality *[2] (urbanization)<br><br>*Subjective:*<br>**ACCESS RECR FACIL**<br>- sufficiently available green spaces in neighborhood<br>**AESTH**<br>- noise from outdoors annoys you<br>- air pollution<br>- bad smells coming from outside<br>- affection by an industry<br>**GENERAL QUALITY**<br>- quality of neighborhood	*X^2 test*<br>- size of municipality **(+)**[m], (0)[f]<br>- no sufficient green spaces in neighborhood **(+)**[m,f]<br>- annoying noise from outdoors (0)[m,f]<br>- air pollution (0)[m,f]<br>- bad smells from outside (0)[m,f]<br>- affection by an industry (0)[m,f]<br>- bad quality of neighborhood (0)[m,f]	*[1] Free time PA categories: [1]none (=sedentary activities), [2]occasional, [3]regular PA, [4]physical training. Categories 2-4 were classified as " doing free time PA"<br>*[2] size of municipality: [1] <10,000 inhabitants, [2] 10,000 – 100,000 inhabitants, [3] > 100,000 inhabitants<br>- for the analyses, reference categories were "municipality size <10,000", a lot or some sufficient green spaces", " no annoying noise from outside", "no bad smells from outside", "no highly polluted air", "no affection by industry" and "good neighborhood environment quality"<br>**RESULTS**:<br>()[m]: specific results for men<br>()[f]: specific results for women
**Bonnefoy et al. 2003**<br><br>**Ref n° 30**	- n = 1,172<br>- "adults"<br>- %F not provided	- random<br><br>- r.r. = 50.2%	Forli, Italy (part of the LARES Study)	CS	*Subjective:*<br>**TOTAL PA**<br>- regular overall PA*[1]	*Objective (inspection sheets)*<br>**ACCESS RECR FACIL**<br>- dwelling proximity to a park (less than 100m vs more than 100m)	*Unknown analysis (unadjusted)*<br>< 100m from a park **(+)**<br>> 100m from a park **(-)**	*[1]PA was categorized into two levels: [1] regularly engage in moderate or intense exercise vs [2] never exercise

**Cochrane et al. 2009**<br><br>**Ref n° 31**	-n= 761<br>- age range: 16 and older<br>-age 15-24: 10%<br>-age 25-44: 36.3%<br>-age 45-64: 30.9%<br>-age 65+: 22.9%<br>- 55% F	- random probability<br><br>-r.r.= 49%	Ten deprived **urban** areas*[1] in Stoke on Trent, England	CS	*Subjective:*<br>(IPAQ; long version)<br>**TOTAL PA**<br>- PA outside work	*Objective:*<br>**TRAFFIC SAFETY**<br>-length of road with moderate traffic levels** (+)<br>- count per km of road of casualties involving public transport (-)<br>**CRIME SAFETY**<br>- count per head of population reporting criminal damage (-)<br>*Subjective*:<br>**ACCESS SERVICES**<br>- walking distance to local convenience store **(-)**<br>- several shops within easy walking distance **(+)**<br>- walking distance to work/place of study **(-)**<br>- walking distance to fast food restaurant **(-)**<br>- how easy to get to supermarket **(-)**<br>**AESTHETICS**<br>- attractive buildings or homes in neighborhood **(+)**	<u>*Multiple linear regression*</u><br>*Objective*:<br>-length of road with moderate traffic levels** **(+)**<br>- count per km of road of casualties involving public transport **(-)**<br>- count per head of population reporting criminal damage **(-)**<br>*Subjective*:<br>- walking distance to local convenience store **(-)**<br>- several shops within easy walking distance **(+)**<br>- walking distance to work/place of study **(-)**<br>- walking distance to fast food restaurant **(-)**<br>- how easy to get to supermarket **(-)**<br>- attractive buildings or homes in neighborhood **(+)**	- Stoke on Trent is a mid-sized conurbation (population approxim. 240,000)<br>*[1] geographical units are called Lower Level Super Output Areas (LSOAs)= smallest units for which population census data are available<br>*[2] PA outside work refers to PA in leisure time, PA for transportation and PA in household and gardening activities. The variable "PA outside work" is square root transformed!<br>*[4] 40 items of perceptions: 17 scored on 5point scale; 23 on 4point scale<br>**within 800m buffer area around OA<br>- correlation between self-reported activity and accelerometer measured activity was moderate to good at 0.57 (N=109)
**Coombes et al. 2010**<br><br>**Ref n° 32**	-n=6,803<br>- *M* age= 51y<br>59% F	Zie p 817<br><br>r.r. = 34%	**Inner city and suburban areas** in Bristol, UK<br><br>Bristol Quality of Life in your Neighborhood Survey 2005 data	CS	*Subjective:*<br>**TOTAL PA**<br>- achieving Chief Medical Officer's (CMO) *[1] guidelines	*Objective:*<br>(ArcGIS 9.2)GIS<br>**ACCESS RECR FACIL**<br>- road distance to nearest green space*[2]<br>**CONNECTIVITY**<br>- n° of junctions per km of road	<u>*Binary logistic regression*</u><br>(crude analyses)<br>- road distance to nearest green space (0)<br>- road density **(+)**→ neg traffic<br>- Aroad density **(+)**→ neg traffic<br>- n° of junctions per km of road **(+)** → pos connectivity	*[1]achieving CMO guidelines = ≥30 min of moderate activity, ≥ 5x/wk<br>*[2] distance by road from the residential location of each respondent to the nearest green space of each type considered. Green spaces were grouped in five categories: [1]Formal; [2]Informal;

						- road connectivity (junctions/cul-de-sacs)<br>- effective walkable area<br>**TRAFFIC SAFETY**<br>- road density<br>- A-road density<br>**LAND USE MIX DIVERSITY**<br>- land use diversity<br>**RESIDENTIAL DENSITY**<br>- % residential buildings in neighborhood	- road connectivity **(+)**<br>- effective walkable area **(+)**<br>- land use diversity (0)<br>- % residential buildings in neighborhood (0)	[3]Natural; [4]Young people's; [5]Sports<br>*^{3}" neighborhood" = area within 800m (~10 minute walk)around the road network from the respondent's home
**De Bourdeaudhuij et al. 2003**<br><br>**Ref n° 33**	- n= 521<br>- *M* age: 41y ± 12.22<br>- 48.3% F<br>-39.3% lived in city center; 54.9% in suburbs; 5.9% countryside	Random sample in Ghent, Belgium<br><br>Questionnaires provided through mail.<br><br>-r.r.= 52.8%	City centre, suburbs & countryside in Ghent, Belgium	CS	*Subjective:*<br>(IPAQ short version last 7 d)<br>**TOTAL WALKING**<br>- minutes of walking<br>**TOTAL PA**<br>- minutes of moderate intensity PA<br>- minutes of vigorous intensity PA	*Subjective**^{1}<br>[1]<br>**RESIDENTIAL DENSITY**<br>- residential density<br>**LAND USE MIX DIVERSITY**<br>- land use mix (diversity uses)<br>**CONNECTIVITY**<br>- connectivity street network<br>**ACCESS SERVICES**<br>- land use mix (access to local shopping)<br>**ACCESS PUBLIC TRANSP**<br>- ease of walk access to public transportation stop<br>**WALKING/CYCLING FACIL**<br>- availability of sidewalks<br>- availability of bike lanes<br>**AESTH**<br>- neighborhood aesthetics<br>**CRIME SAFETY**<br>- perceived safety from crime<br>**TRAFFIC SAFETY**<br>- perceived safety from traffic<br>**ACCESS RECR FACIL**<br>-convenience of PA facilities	*Multiple regression analyses*<br>*(Adjusted for demographics)*<br>*MEN:*<br>- residential density (0)^{1,2,3}<br>- land use mix (diversity uses) (0)^{1,2,3}<br>- land use mix (access to local shopping) (0)^{1,2,3}<br>- ease of access to public transportation stop (0)^{1,2,3}<br>- availability of sidewalks **(+)**^{1} (0)^{2,3}<br>- availability of bike lanes (0)^{1,2,3}<br>- neighborhood aesthetics (0)^{1,2,3}<br>- perceived safety crime (0)^{1,2,3}<br>- perceived safety traffic (0)^{1,2,3}<br>- connectivity street network (0)^{1,2,3}<br>-convenience of PA facilities **(+)**^{3} (0)^{1,2}<br><br>*WOMEN:*<br>- residential density (0)^{1,2,3}<br>- land use mix (diversity uses) **(+)**^{1} (0)^{2,3}<br>- land use mix (access to local shopping) **(+)**^{2} (0)^{1,3}<br>- ease of access to public transportation stop **(+)**^{1} (0)^{2,3}	*^{1} questionnaire [1] contains items believed to be related to walking and cycling for transportation and leisure (40 items); questionnaire [2] assessed environmental factors believed to be related mainly to recreational PA (41 items)<br>- validity and reliability environmental questionnaire [1]: (obj interrater reliability .80 -.90 = high to very high)<br>→ validity between .21 and .91<br>→ reliability between .40 and .97<br>- logarithmic transformations were used to improve normality of distribution for the PA variables (walking, mod, and vig PA)<br><br>- **RESULTS**:<br>()^{1} = related to min of walking<br>()^{2} = related to min of moderate PA<br>()^{3} = related to min of vigorous PA

							- availability of sidewalks (0)[1,2,3]<br>- availability of bike lanes (0)[1,2,3]<br>- neighborhood aesthetics (0)[1,2,3]<br>- perceived safety crime (0)[1,2,3]<br>- perceived safety traffic (0)[1,2,3]<br>- connectivity street network (0)[1,2,3]<br>-convenience of PA facilities **(+)**[3] (0)[1,2]	
**De Bourdeaudhuij et al. 2005**<br><br>**Ref n° 34**	-n = 526<br><u>Belgium</u>:<br>-n=279<br>-*M* age: 37.2 ± 12.3<br>65.9% F<br><br><u>Portugal:</u><br>-n = 247<br>-*M* age: 35.1 ±11.5<br>64.5% F	Convenience sampling through worksites, libraries and socio-cultural societies	Ghent, Middle European city (BEL) and Oeiras, a Southern European city (POR)	CS	*Subjective*<br>(IPAQ long version **usual** week)<br><br>- MVPA at work<br>- cycling transportation<br>- walking transportation<br>- all active transport<br>- MVPA in garden<br>- walking leisure time<br>- mod PA leisure time<br>- vig PA leisure time<br>- all MVPA leisure time<br>- total PA at least mod<br>- vig PA 20 min<br>- mod PA 30 min	*Subjective**<br>**RESIDENTIAL DENSITY**<br>- residential density<br>**LAND USE MIX DIVERSITY**<br>- land use mix (diversity uses)<br>**CONNECTIVITY**<br>- connectivity street network<br>**ACCESS SERVICES**<br>- land use mix (access to local shopping)<br>**ACCESS PUBLIC TRANSP**<br>- ease of walk access to public transportation stop<br>**WALKING/CYCLING FACIL**<br>- availability of sidewalks<br>- availability of bike lanes<br>**AESTH**<br>- neighborhood aesthetics<br>**CRIME SAFETY**<br>- perceived safety from crime<br>**TRAFFIC SAFETY**<br>- perceived safety from traffic<br>**ACCESS RECR FACIL**<br>-convenience of PA facilities	<u>*Independent samples t-tests*</u><br>- country*[1] **(+)**[1,2,5,7,10,11,12] **(-)**[3] (0)[4,6,8,9] → not taken up in summary calculation<br><br><u>*Multiple regression analysis (adj for A,G,E)*</u><br><u>*PORTUGAL*</u>:<br>- residential density (0)<br>- land-use mix diversity **(+)**[4] (0)[6,9,10]<br>- land-use mix access (to local shopping) (0)<br>- ease to walk to public transportation stop (0)<br>- availability of sidewalks **(+)**[6] (0)<br>- availability of bike lanes (0)<br>- neighborhood aesthetics (0)<br>- perceived safety crime (0)<br>- perceived safety traffic (0)<br>- connectivity street network (0)<br><br><u>*BELGIUM*</u>:<br>- residential density (0)<br>- land-use mix diversity **(+)**[4,6,9,10]<br>- land-use mix access (to local shopping) (0)<br>- ease to walk to public transportation stop (0)	*[1] country = Portugal= reference category ; level of significance 99%<br>- before running multiple linear regressions, bivariate correlations were calculated. If corr >0.50 between two predictors: only predictor with highest bivariat corr with criterion was kept<br><br>**RESULTS**:<br>()[1] related to MVPA at work<br>()[2] related to cycling transportation<br>()[3] related to walking transportation<br>()[4] related to all active transport<br>()[5] related to MVPA garden<br>()[6] related to walking leisure<br>()[7] related to mod PA leisure<br>()[8] related to vig PA leisure<br>()[9] related to all MVPA leisure<br>()[10] related to total PA at least mod<br>()[11] related to vig PA 20 min<br>()[12] related to mod PA 30 min<br>!! Only variables 4,6,9 and 10 were used for multiple regression analyses

							- availability of sidewalks (0)<br>- availability of bike lanes (0)<br>- neighborhood aesthetics (0)<br>- perceived safety crime(0)<br>- perceived safety traffic (0)<br>- connectivity street network (0)<br>- worksite environment (0)<br>- PA supplies in home environment **(+)**[9,10] (0)[4,6]<br>- convenience of PA facilities (0)<br>- satisfaction with neighborhood services (0)<br>- emotional satisfaction with neighborhood (0)	
**de Geus et al. 2008**<br><br>**Ref n° 35**	-n=343 adults<br>- age range: 18-65<br>-*M* age cyclists= 41.9y ±9.5<br>- *M* age non-cyclists= 40.43y ±8.8<br>- 57%F	- non-random recruitment through announcement in newsletter of National Health Surveillance company<br><br>-r.r. : unknown, due to the indirect way of recruitment	Flanders, Belgium*[1]	CS	*Subjective:*<br>(Online) questionnaires<br>**CYCLING TRANSPORT**<br>Cycling for transport*[2]	*Subjective*<br>**URBANIZATION**<br>- living area (town of <30,000 inhabitants or town of > 30,000 inhabitants)<br>**ACCESS SERVICES**<br>- destinations **(minutes)**:<br>- food shops<br>- other shops<br>- work<br>**ACCESS PUBLIC TRANSP**<br>- bus, tram or metro stop<br>**TRAFFIC SAFETY**<br>**WALKING/CYCLING FAC**<br>**CRIME SAFETY**<br>- traffic variables in the neighborhood:<br>- traffic danger<br>- bicycle lanes<br>- crime<br>- traffic safety<br>- traffic variables on the road to work:<br>- traffic danger<br>- bicycle lanes<br>- crime	Cyclers (C) vs non-cyclers (NC)<br>*X^2 tests*<br>- living outside a big city*[3] **(-)**<br><br>*Independent t tests:*<br>- destinations:<br>- food shops **(-)**<br>- other shops **(-)**<br>- work **(-)**<br>- bus, tram or metro stop (0)<br>- traffic variables in the neighborhood<br>- traffic danger (0)<br>- bicycle lanes (0)<br>- crime (0)<br>- traffic safety (0)<br>- traffic variables on the road to work:<br>- traffic danger (0)<br>- bicycle lanes (0)<br>- crime (0)	*[1]Participants had to be living at max. 10 km from their workplace<br>*[2] cycle at least once a week to work in the last 6 months prior to the start of the study (➔ cycling group)<br>*[3] big city: ≥30,000 inhabitants<br>-traffic variables included traffic danger, condition and presence of cycle lanes, crime and traffic safety (+ p 705)<br>- environmental destinations are referring to the travel time in minutes!

**Dygryn et al. 2010**<br><br>**Ref n° 36**	-n= 70<br>- *M* age= 33.3y ±13.7<br>- 41.4% F	- random<br>-r.r.= 51.85%	Olomouc, Czech Republic;<br><br>Data collection **spring** 2009	CS	*Objective:*<br>**TOTAL PA**<br>- average daily steps (Yamax SW-700 pedometer worn for 7 days)	*Objective:*<br>**WALKABILITY**<br>- neighborhood walkability (using GIS: index based upon residential density, connectivity, land-use mix and floor retail area)	Two sample t-tests:<br>Weekdays:<br>- living in high walkable area **(+)**<br>Weekend days:<br>- living in high walkable area **(+)**<br>Whole week:<br>- living in high walkable area **(+)**	- walkability indexes were calculated for every individual participant after recruitment<br>- no definitions of "neighborhood" and no definitions of "low" and "high" walkability → only reference Frank et al. and Cerin et al.
**Ellaway et al. 2005**<br><br>**Ref n° 37**	- n = 6,919<br>- age range: 18-65y	- Cities: purposeful<br>- Individuals: random<br><br>- r.r. not provided	LARES study in 8 European countries<br><br>Data collection 2002-2003	CS	*Subjective:*<br>**TOTAL PA**<br>-"frequent PA"	*Objective:*<br>(inspection sheets)<br>**AESTH (**<br>- amount of greenery*[1]<br>- amount of incivilities (litter, graffiti and dog mess)	*Logistic regression analysis (adj for A, G, SES, and city of residence)*<br><br>- amount of greenery **(+)**<br>- amount of incivilities **(-)**	Data collected in Angers (France); Bonn (Germany); Bratislava (Slovakia); Budapest (Hungary); Ferreira do Alentejo (Portugal); Forli (Italy); Geneva (Switzerland) and Vilnius (Lithuania)<br>- PA recoded in 2 levels: (never vs seldom/often)<br>*[1] amount of greenery → greenery visible on the dwelling and streets immediately surrounding it<br>- incivilities and greenery recoded in 5 levels (low=1 to high=5)
**Engbers & Hendriksen 2010**<br><br>**Ref n° 38**	- n = 799<br>- *M* age = 41.2y ±11.0<br>- 49.6% F	- random<br><br>- r.r. = 39.6%	The Netherlands,<br><br>Data collection spring 2008	CS	*Subjective:*<br>(internet survey SQUASH questionnaire)<br>**CYCLING TRANSPORT**<br>- cycling to work (commuter cycling)	*Subjective:*<br>(internet survey)<br>**ACCESS SERVICES**<br>- living distance to work	*Univariate logistic regression: (adj for A, G, E and income)*<br><br>- living distance to work ≤8km **(+)**	- SQUASH questionnaire covers 14 specific PA behaviors, including cycling and walking to work
**Foster et al. 2004**<br><br>**Ref n° 39**	-n = 4,175<br>- *M* age= 43y ± 15<br>- 57.1% F	- addresses: multi-stage cluster random probability design<br>- individuals: Randomly<br><br>- r.r. = 64% of baseline in wave 1 (52% r.r. in this wave)	United Kingdom, Active for Life campaign,<br>Data collection all in "wave 2"; 1996	CS	*Subjective*<br>Self reported walking behavior past 4 weeks:<br>**TOTAL WALKING**<br>- at least 15min/wk:<br>- at least 150min/wk:	*Subjective: all **high vs low***<br>**SAFETY**<br>- safety of walking alone during the day<br>- safety of walking alone during night<br>**ACCESS RECR FACIL**<br>- convenience of parks/open spaces<br>**ACCESS SERVICES**<br>- convenience of local shops*<br>**AESTH**<br>- neighborhood aesthetics<br>**TRAFFIC SAFETY**	*2 x 2 tables:*<br>Walking ≥ 15min/wk:<br>No significance for men, nor women<br>Walking ≥ 150min/wk:<br>- safety walking alone day (0)[2] (0)[1]<br>- convenience parks/open spaces (0)[1] (0)[2]<br>- other variables: no significance for men, nor women	- Walking included any occasion of walking for at least 15min, whatever the purpose of the walk<br>- * "convenience" refers to the destination being at walking distance<br><br>**RESULTS**:<br>()[1] = specific results for men<br>()[2] = specific results for women

						- neighborhood traffic levels<br>- access to leisure centre		
**Foster et al. 2009**<br><br>**Ref n° 40**	- n = 13,927<br>- age range = 45y – 74y<br>- mean age:<br>MEN: 62y<br>WOMEN: 61y<br>- 56% F	- multi-stage cluster random probability sampling (City of Norwich, people living ≤9km from city center)	Urban and urban fringe area of Norwich<br>UK, EPIC-Norfolk cohort<br><br>Data collection<br>Crime audit 2000	CS	*Subjective:*<br>(EPAQ2)<br>**LTPA**<br>- swimming for recreation<br>- facility based PA (FBPA)*[1]<br>**CYCLING RECR**<br>- cycling for recreation<br>**WALKING RECR**<br>- walking for recreation	*Objective:*<br>(GIS, sec data sets and environmental audits)<br>**ACCESS RECR FACIL**<br>- proximity to nearest swimming pools<br>- proximity to nearest sports centers and facilities<br>- proximity to nearest public open/green space<br>**TRAFFIC SAFETY**<br>- local proximity to traffic levels (road traffic volume index scores (RTVIS))<br>**CRIME SAFETY**<br>- neighborhood area levels of crime (ward level data)	*Multiple regression analyses*<br>*(adj for A, social status, E, car use, area deprivation, self reported health, mode of travel to work and occupational PA)*<br><br>**Swimming for recreation**<br>- prox nearest public swimming pool (0)[1,2]<br>- prox nearest public or private swimming pool **(-)**[2], (0)[1]<br>FBPA<br>- nearest public adults education sports center (0)[1,2]<br>- nearest public sports center (0)[1,2]<br>- nearest public or private sports center (0)[1,2]<br>Walking for recreation *[2]<br>- nearest public park (0)[1,2]<br>- nearest nature reserve (0)[1,2]<br>- nearest river walk (0)[1,2]<br>- nearest any green space (0)[1,2]<br>Walking for recreation<br>- crime level (0)[1,2]<br>Cycling for recreation<br>- traffic (lowest level = ref cat)<br>- traffic **(-)**[1,2]	- residential location of each participant was determined using their postcode<br>- EPAQ2 = EPIC PA questionnaire: activities during the past year + frequency (none; <1x/month; 1x/month; 2-3x/month; 1x/wk; 2-3x/wk; 4-5x/wk; ≥5x/wk)<br>*[1] FBPA includes activities like aerobics, exercise with weights, badminton, and yoga<br>- *for the analyses, "none" category of the PA was set as the ref cat*<br>**RESULTS**:<br>()[1]: specific results for men<br>()[2]: specific results for women<br><br>*[2]sample of 6,214 adults (56%F)<br>- !! negative Ors in the "proximity" outcome means: further distance (= less proximity) → less swimming for recreation
**Foster et al. 2011**<br><br>**Ref n° 41**	- n = 13,927<br>- *M* age=62.1y ±9.1<br>- age range = 45y – 74y<br>- 56%F	- multi-stage cluster random probability sampling<br>- r.r. = 88%	UK, EPIC-Norfolk cohort<br>Data collection<br>PA: 1998-2000	CS	*Subjective:*<br>**CYCLING RECREATION**<br>- leisure cycling<br>**CYCLING TRANSPORT**<br>- cycling to work (commuter)	*Objective:*<br>(GIS)<br>**TRAFFIC SAFETY**<br>- road traffic volume 0.5km<br>- road traffic volume 1 km<br>- road traffic volume 2 km<br>- road traffic volume 3.2 km	*Logistic regression*<br>*(adj for *)*<br>MEN: positive evolution: less traffic, more cycling<br>WOMEN: see "men"<br><br>This both for cycling for recreation as for commuter cycling	- four distance based buffers around individual's postcodes were calculated by GIS; summation of total length 4 road types within buffers and weighted based on av. road speed for each classification. Quartiles were used, lowest quartile, light traffic= reference category!<br>* leisure cycling models adj for age

								,social status, E, car ownership, travel mode to work and occupational PA; commuter cycling models adj for age, social status, car ownership, area deprivation, occupational PA and recreational PA
**Frömel et al. 2009**<br><br>**Ref n° 42**	- n= 9950<br>- age range = 24y – 64y<br>- *M* age= 42.2y ±10.7<br>- 50.6% F	- sampling location: Systematic random sampling<br>- individuals: randomly<br><br>- r.r. = 58%	National study in Czech Republic, developing country	CS	*Subjective*<br>(IPAQ, short version, last 7 days)<br>**TOTAL PA**<br>- meeting guidelines for vig PA (≥3x≥20min/wk)<br>- meeting guidelines for mod PA (≥5x≥30min/wk)<br>**TOTAL WALKING**<br>- meeting guidelines for walking (≥5x≥30min/wk)<br>**LTPA**<br>- participation in organized sports	*Subjective*<br>**URBANIZATION**<br>- town size*[1]	*Binary logistic regression*:<br>Meeting guidelines vigorous PA<br>Town size **(-)**[1,2]<br>Meeting guidelines moderate PA<br>Town size **(-)**[1,2]<br>Meeting guidelines walking<br>Town size (0)[1,2]<br><br>*Unpublished analyses*<br>Participation in organized sports<br>- small town (39%) vs bigger town and big town (24%)<br>More participation in small town	- analyses were performed for males and females separately<br>*[1] "town size" was indicated by respondents as the n° of residents in their town from >100,000 to <1,000 and was categorized as [1] large city: >100,000; [2] bigger town: 30,-100,000; [3] small town: 1,-29,999 and [4] small village: <1,000.<br>*[2] large city = reference category<br><br>**RESULTS:**<br>()1= specific results for men<br>()2 = specific results for women
**Gast et al. 2007**<br><br>**Ref n° 43**	- n = 18,695<br>- age range = 20y - 69y	Each year random sample<br><br>- r.r. = ±50%	The Netherlands, NethHIS/POLS (Health Interview Survey)<br><br>Data collection 1981 - 2004	LONG	*Subjective:*<br>(self-administered questionnaires, ao SQUASH)<br>- **LTPA**:<br>walking, bicycling, gardening and playing sports	*Objective:*<br>**URBANIZATION**<br><br>- urbanization	*Trend in LTPA across urbanization degrees:*<br><br>Period 1990-1997:<br>- increase in LTPA (time spent) across years, no differences across urbanization degrees<br><br>Period 2001-2004:<br>- no evolution trend in LTPA (time spent) across years, no differences across urbanization degrees	- participation in sports/other forms of PA → if yes: identification of most common activities (maximal 4)+ frequency in last 2 weeks<br>- urbanization scale ≈ "address density" within 1 km of an address. (High: ≥ 1500 addresses/km²; moderate: 1000-1500 addresses/km²; low ≤ 1000 addresses/km²)<br>- because of a split in trend in mean time spent on LTPA between period 1990-1997 and 2001-2004, due to the use of two different questionnaires, LTPA levels were evaluated separately for these two periods.

**Gidlöf-Gunnarsson & Öhrström 2007**<br><br>**Ref n° 44**	- n = 500<br>- age range: 18y – 75y<br>- *M* age = 43.6 ±15.18<br>- 56% F	Clusters: purposeful<br>Individuals: random<br><br>- r.r. = 59%	Urban residential settings<br>Stockholm & Göteborg, Sweden	CS	*Subjective:*<br>**TOTAL PA**<br>- walking/exercising in neighborhood every day/one/few times/wk	*Subjective:*<br>**ACCESS RECR FACIL**<br>- availability green areas (poorer access vs better access)	*Unknown analysis*<br>NOISE-NOISE<br>- availability green areas **(+)**<br>NOISE-QUIET<br>- availability green areas **(+)**<br><br>(noise-noise condition residents benefit most from availabililty to green areas)	- participants were divided into two groups: noise/noise (n=133) and noise/quiet (n=367) (the latter had access to a quiet side of the residence); the domination noise source was road traffic<br>- green area = area in city plans with green surface, trees, …<br>- for the analyses, poorer access is set as "reference"
**Guthold et al. 2008**<br><br>**Ref n° 45**	- n European countries = 27,800<br><br>- *M* ages European countries = 40.0y – 45.5y<br><br>- % F in European countries = 50.1-62.8%	Random national samples, using a multistage cluster design<br><br>- r.r. total sample: 81.7%<br><br>- r.r. European countries: 39.7% - 98.5%	51 countries **worldwide**, 12 European (World Health Survey)<br><br>Data collection 2002-2003	CS	*Subjective:*<br>(interview IPAQ, short form)<br>**TOTAL PA**<br>- meeting PHRs	*Objective:*<br>**URBANIZATION**<br>- urbanization (urban vs rural)	*Unknown analysis*<br>Less physical **IN**activity in rural areas<br><br>So higher PA levels in rural areas compared to urban<br><br>(this finding counts for all 51 countries (so also non-European))	- European countries involved were Bosnia-Herzegovina; Croatia; Czech Republic; Estonia; Georgia; Hungary; Russian Federation; Slovakia; Slovenia; Spain; Turkey and Ukraine<br>- not meeting criteria (≥3x ≥20' vig/wk or ≥5x≥30' mod or walk/wk or ≥5x≥600METmin/wk walk, mod, vig) is considered **inactive**<br>- % of European people living in urban areas ranged from 44.6% (Bosnia-H) to 88.0% (Russian Fed)
**Harrison et al. 2007**<br><br>**Ref n° 46**	-n = 15,461<br>- *M* age= 49.8y ± 17.6<br>- 54.8% M	Systematic random sampling:individuals<br><br>-r.r.= 70.1%	**Two districts** in Northwest England, UK	CS	*Subjective*<br>(Godin and Shephard instrument; last week)<br>**TOTAL PA**<br>- being physically active *[1]	*Subjective*:<br>**ACCESS PUBLIC TRANSPORT**<br>- well-placement of home for public transport<br>**ACCESS SERVICES**<br>- well-placement of home for general shopping<br>**ACCESS RECR FACIL**<br>- well-placement of home for leisure facilities<br>**TRAFFIC SAFETY**<br>- problem of speeding traffic<br>**CRIME SAFETY**<br>- problem of vandalism<br>- problem of assaults/muggings<br>- subject of crime past year	*Modified Poisson regression*<br>- well-placement of home for public transport (0)<br>- well-placement of home for general shopping (0)<br>- well-placement of home for leisure facilities **(+):**<br>*very well (ref)*<br>*fairly well (0)*<br>*average (0)*<br>*not very well* **(-)**<br>*badly* **(-)**<br>- problem of vandalism (0)<br>- problem of assaults/muggings (0)<br>- problem of speeding traffic **(+):**	Postal codes were linked to area deprivation<br>PIMs= population impact measures: provide information on incidence to estimate the number of people in a total population who may benefit (or be at risk) from an intervention<br>*[1] being physically active= participating in at least 5 sessions/wk of moderate or vigorous PA, with each session lasting ≥15 min

						**SAFETY**<br>- feelings of safety in neighborhood day<br>- feelings of safety in neighborhood night	*not a problem (ref)*<br>*some problem* **(+)**<br>*serious problem (0)*<br>- subject of crime past year **(+)**<br>- feelings of safety in neighborhood day (ref= yes) **(-)**<br>- feelings of safety in neighborhood night (ref=yes) **(-)**	
**Hillsdon et al. 2006**<br><br>**Ref n° 47**	- n= 4,732<br>- age range: 45y-74y	Purposeful through GPs	City of Norwich, England<br>Participants were part of the EPIC* study<br>Data collection PA: 1997-1999<br>Data collection green space:2005	CS	*Subjective*:<br>(activities past year)<br>**LTPA**<br>- total hours of recreational PA/wk	*Objective:*<br>(GIS)<br>**ACCESS RECR FACIL**<br>- access to open green space*[1]<br>- distance to green space from home residence	<u>*Multiple regression models*</u><br>*(covariates: A, G, area deprivation, E, ethnicity and distance to city boundary*[3]*)*<br>- distance to open green space (0)	*[1]EPIC (European Prospective Investigation of Cancer), Norfolk cohort<br>- PA was assessed through providing 36 different activities and asking the participants to give average time spent for these activities in the last year and average number of times they had been performed<br>- PA levels were logarithmically transformed to treat their skewed distribution<br>*[2]61 green spaces (≥2 hectares & open for public access) in Norwich were surveyed using a 69-item quality audit tool, based upon 8 neighborhood themes: accessibility, maintenance, recreational facilities, amenity provision, signage and lighting, landscape, usage, and atmosphere.<br>*[3] access to city boundary was included to control for those who were living closer to the countryside (more access to countryside green)
**Jones et al. 2009**<br><br>**Ref n° 48**	- n = 6,821<br>- *M* age= 51y<br>- 59% F	Clusters: purposeful<br>Individuals: Random<br><br>- r.r. = 34%	Bristol, England, UK<br>Data collection 2005	CS	*Subjective:*<br>**TOTAL PA**<br>- achieving PA guidelines (= mod PA ≥5x/wk)	*Objective:*<br>(GIS)<br>**ACCESS RECR FACIL**<br>- distance to the nearest greenspace<br>*Subjective:*<br>**ACCESS RECR FACIL**	<u>*Logistic regression modeling*</u><br>*Objective:*<br>*(adj for A, G, self-rated health)*<br>MOST AFFLUENT IMD QUARTILE<br>- distance nearest gr.space (0)<br>- distance formal gr.space (0)<br>- distance informal gr.space(0)	- neighborhoods were defined by GIS as 800m surrounding the centroid of a postcode address<br>- objective measures of greenspace included 5 types: formal, informal, sports, natural and youth people’s<br>- IMD = Index of Multiple Deprivation

						- greenspace access<br>**SAFETY**<br>- greenspace safety	- distance natural gr.space(0)<br>- distance young people's gr.space(0)<br>- distance sports gr.space(0)<br>- greenspace access **(-)**<br>- greenspace safety **(-)**<br><br>MOST DEPRIVED IMD QUARTILE<br>Same results, but stronger gradient for safety perceptions and visit frequency compared to most affluent IMD quartile	- more deprived neighborhood residents had an overall better access to objectively measured greenspace and to types "informal, natural and young people's"; for greenspaces of type "formal" and "sports" the reverse was true<br>- for perceptions of access and safety (X^2tests): less deprived perceived best access, most deprived perceived least access and smaller visit frequency/least active!<br>- for the analyses, the lowest quartiles were set as the reference categories (i.e. "smallest distance" for the objective measures and "very easy/safe" for the perceptions)
**Kamphuis et al. 2007**<br><br>**Ref n° 49**	- n= 3,839<br>- M age= 47.69y±13.11<br>- 52.5% F	-clusters: purposeful<br>- individuals: random<br>- r.r.=64.4%	177 neighborhoods in Eindhoven, 5th largest city in the Netherlands<br><br>GLOBE study October 2004	CS	*Subjective*:<br>(SQUASH questionnaire)<br>**LTPA**<br>- sports participation*[1](no/yes)	*Subjective*:<br>**SAFETY**<br>- safety of neighborhood<br>**AESTH**<br>- attractiveness of neighborhood<br>**ACCESS RECR FACIL**<br>- availability of facilities in neighborhood (**in**sufficient)<br>**CRIME SAFETY**<br>- social disorganization of neighborhood*[2]	*Multilevel logistic regressions:*<br>*(adj for A, G, E and country of origin)*<br>Doing **NO** sports:<br>- unsafe neighborhood **(+)**<br>- unattractive neighborhood **(+)**<br>- insufficient places for PA (0)<br>- social disorganization (0)	*[1] sports participation was asked as "doing any moderate- or high-intensity sports at least once a week"<br>- model2: neighborhood attractiveness + neighborhood safety + social network + social cohesion<br>*[2] "social disorganization" proxies: litter, graffiti, vandalism, hassling of people on streets, drunken people on streets; variable "social disorganization is divided into tertiles low, medium, and high<br>- clustering of sports participation was determined by MOR (median Ors) MCMC procedure in MLWin<br>- clustering of sports participation in neighborhoods (neighborhood attractiveness+ neighborhood safety+social network+social cohesion)
**Keijer & Rietveld 2000**	- n = 82,835<br>- adults	Unknown sampling	National Travel Survey The Netherlands	CS	*Subjective:*<br>**WALKING TRANSPORT**<br>- walking to/from	*Subjective:*<br>**ACCESS PUBLIC TRANSP**<br>- distance to/from railway	*Unknown analysis*<br>Walking to/from railway station<br>- distance to/from railway	- home-end = the travelled path between the home residence and the railway station

**Ref n° 50**			Data collection 1994		railway station<br>**CYCLING TRANSPORT**<br>- cycling to/from railway station	station	station **(-)**<br><u>Cycling to/from railway station</u><br>- distance to/from railway station **(-)**<br>**At the home-end:**<br>- living ≤ 1.5 km: walk<br>- living between 1.5 and 3.5km: cycle<br>- living further: motorized (PT)<br>**At the activity-end:**<br>- distance to destination < 2km: walk<br>- more distant: motorized (PT)<br>- bicycle plays the biggest role at the home-end	- activity-end = the travelled path between the railway station and the place of the destination<br>- PT = public transport
**Kwasniewska et al. 2010**<br><br>**Ref n° 51**	- n= 7,280 (3747 M; 3533 F)<br>- *M* age= 38.0y±11.18<br>- 48.5% F	- urban areas: Purposeful (strata)<br>- individuals: random<br><br>- r.r. M= 74.3%<br>- r.r. F= 79.3%	Poland:<br>2 rural, 2 small urban and 2 large urban areas<br><br>National Multicentre Health Study: WOBASZ Project October 2004-March 2005	CS	*Subjective:*<br>**GENERAL ACT TRANSP**<br>- commuting PA<br>- 0min walk/cycle<br>- 1-14min walk/cycle<br>- 15-29min walk/cycle<br>- ≥30min walk/cycle	*Objective*<br>**URBANIZATION**<br>- rural (≤ 8,000 inhabitants)<br>- small urban (8,000-40,000 inhabitants)<br>- urban (> 40,000 inhabitants)	<u>*Logistic regression analysis*</u><br>*(adj for age, education, place of residence, income, smoking and other domains of PA)*<br><br>**→ activity (-) with urbanization**<br><br>Same results men and women	- exclusion of: housewives; unemployed; retired<br>- inclusion of individuals working/studying outside of home<br>* ref category = rural
**Maas et al. 2008**<br><br>**Ref n° 52**	- n= 4,899<br>- *M* age= 46.73y<br>- 54.4% F	- DNGSP-2: 104 GPs, random sample (clusters: purposeful, individuals random)	The Netherlands<br><br>Data gathered from two studies:<br>- DNGSP-2, 2001 →PA<br>- LGN4, 2001 → environmental data (25x25m grit cells in the whole of the Netherlands)	CS	*Subjective:*<br>SQUASH questionnaire:<br>**TOTAL PA**<br>- meeting public health recommendations PA (5x30')<br>**WALKING RECR**<br>- walking leisure<br>**WALKING TRANSP**<br>- walking commuting (only if job/school)<br>**CYCLING RECR**<br>- cycling leisure<br>**CYCLING TRANSP**<br>- cycling commuting	*Objective:*<br>**ACCESS RECR FACIL**<br>- % green space within 1km radius around postal code coordinates*[1]<br>- % green space within 3km radius around postal code coordinates	<u>*Multilevel logistic regression (controlled for A,G, E, income and urbanicity)*</u><br><u>*Poisson model*</u>:<br><u>Meeting public health recommendations PA:</u><br>- % green 1 km (0)<br>- % green 3 km (0)<br><u>Other PA domains:</u><br>-participation<br>-% green 1km **(+)**[1] **(-)**[3,4,6] (0)[2,5]<br>- % green 3 km **(-)**[3](0)[1,2,4,5,6]<br>- time spent/wk*[2]<br>- % green 1km **(+)**[1,6] **(-)** (0)[3,4,5]<br>- % green 3 km **(+)**[1,6] **(-)**[3] (0)[4,5]	*[1] postal codes consist of 6 numbers and the same six character postal code is shared by no more than about 15-20 households)<br>- Urban areas are considered to have limited green space + high availability of facilities at walking and cycling distance/ rural: more green, less availability of facilities<br>- green space: 25 x 25m grid domin.<br>- green space= urban green space, agricultural green space, forests and nature conservation areas<br>- !!urbanization: [1] very highly urban (score1!); [2] highly urban; [3]

					(only if job/school)<br>**LTPA**<br>- sports<br>- gardening (only those with a garden)		Age-specific differences!(**marked in red**)<br>- *walking leisure*: strongest neg relationship for 12-25y, then >65, and least strong for 26-65y!<br>- *cycling leisure:* strongest neg relationship for 12-17y<br>- *cycling comm*.: strongest >65y<br>- *gardening*: strongest >65 and 17-25y<br>*Interaction effects green space and urbanicity*<br>Green space<br>Strongest positive relationship agricultural green space and PA<br>Urbanicity (!!)<br>Strongest positive relationship slightly urban areas and PA<br>Urbanicity (+)	moderately urban; [4] slightly urban; [5] non-urban (score 5!)<br>- urbanicity was strongly positively related to the total % of green space (r=.60); strongly positively related to % agricultural green space (r=.64); negatively related to % urban green space (r= -.42); much smaller corr with # natural green space<br>*****[2]if "yes" on being physically active, what was the relationship between time spent per week on this type of PA and the % of green space<br>** if no subdivision is made, both 1km and 3km are having the same symbol (+, 0 or -)<br>**RESULTS**:<br>()[1] specific results for gardening<br>()[2] specific results for sports<br>()[3] specific results for walking leisure<br>()[4] specific results for cycling leisure<br>()[5] specific results for walking comm<br>()[6] specific results for cycling comm
**Maat & Timmermans 2009**<br><br>**Ref n° 53**	- n = 1,094<br>- *M* age= 43.4y<br>- 52.6% F	Unknown sampling method (national travel survey)	Randstad (Amsterdam-Utrecht) region, The Netherlands<br><br>Data collection 2000	CS	*Subjective:*<br>**CYLING TRANSP**<br>- slow transport (=cycling) for commuting purposes (ref cat = car)	*Objective*:<br>**RES DENSITY**<br>**ACCESS PUBLIC TRANSP**<br>**ACCESS SERV**<br>- residential density<br>- distance from home to the railway station<br>- commuting distance	Multinomial logit models:<br>*MEN*:<br>- residential density **(+)**[1] (0)[2,3]<br>- distance home-railway (0)[1,2,3]<br>- commuting distance **(-)**[1] (0)[2,3]<br><br>*WOMEN*:<br>- residential density (0)[1,2,3]<br>- distance home-railway (0)[1,2,3]<br>- commuting distance **(-)[1,2,3]**<br>- distance to work station **(-)[2]** (0)[1,3]	- urban density index = total density of housing, jobs and retail floor space<br>- groups were divided based upon car ownership: single-earner households, dual-earner households and dual-earner households with one car<br>- ()[1] = specific results for single-earner households<br>- ()[2] = specific results for dual-earner households<br>- ()[3] = specific results for dual-earner households with one car
**Mason et al. 2011**	- n= 5,657<br>- age: ≥18y<br>- 60% F	Stratified (neighborhoods) Random	23 deprived neighborhoods Glasgow, UK	CS	*Subjective:*<br>Walking behavior<br>**TOTAL WALKING**	*Objective*<br>**URBANIZATION**<br>- location type of the area*	*Bivariate analyses*<br>Location type of the area **(+)**<br>- inner suburbs	*- neighborhoods were subdivided based on significant boundaries or concentrations of contrasting built

**Ref n° 54**		(individuals)<br><br>- r.r.= 50.3%			- "NW5": walking in neighborhood for 5 or more days/wk	*Subjective*<br>- human capital → amenity **use** (within neighborhood or elsewhere)<br>**ACCESS TO RECR FACIL**<br>- sport facilities<br>- parks/play areas<br>**ACCESS TO SERVICES**<br>- post office<br>- small/local grocer<br>- supermarket<br>- general shops (non-food)<br>- social venues<br>- library<br>- community center<br>- job center<br>- environmental capital:<br>**AESTH**<br>- attractiveness buildings in neighborhood<br>- attractiveness environment<br>- tranquility of environment<br>- quality of parks/open spaces<br>- social and community capital<br>**SAFETY**<br>- feeling of safety walking alone at night	- peripheral estates *1.5x morelikely to achieve NW5 than inner-city and inner-suburbs*<br><u>Human capital</u><br>- sports facilities **(+)**<br>- parks/play areas **(+)**<br>- post office (0)<br>- small/local grocer (0)<br>- supermarket (0)<br>- general shops (non-food) **(+)**<br>- social venues **(+)**<br>- library **(+)**<br>- community center (0)<br>- job center (0)<br><u>Environmental capital</u><br>- attractiveness buildings (0)<br>-attractiveness environment (0)<br>- tranquility environment (0)<br>- quality parks/open spaces **(+)**<br><u>Social and community capital</u><br>- feelings safety walking alone after dark **(+)**	forms. Three types are represented: [1]inner-city mass housing estates (reference cat); [2] inner suburbs; [3] peripheral estates<br>- walking was assessed by asking "in a typical week, on how many days do you go for a walk around the neighborhood?"<br>- neighborhood was defined as "5-to-10 min walk around the home"<br>- !! only frequency, not duration of the trips, was assessed<br>- 10.0 % of the variance was explained at the neighborhood level
**Miles et al. 2008**<br><br>**Ref n° 55**	- n = 2,123<br>- mean age = 48y<br>- 65% F	LARES study	7 European cities in France, Germany, Hungary, Slovakia, Italy, Switzerland and Lithuania (LARES study)<br><br>Data collection 2001 -2002	CS	*Subjective:*<br>**LTPA**<br>- sports/physical exercise*	*Objective:*<br>(direct observation)<br>**AESTH**<br>- neighborhood physical disorder **<br>**TRAFFIC SAFETY**<br>- traffic volume<br>**RES DENSITY**<br>- residential density<br>*Subjective*:<br>(face-to-face)	<u>*Multinominal logistic regression and relative risk ratios (RRR):*</u><br>*(adj for A, G, E, M, disability status, years lived in neighborhood, household size, tenure of dwelling)*<br>*MEN*:<br>- neighborhood disorder (0)<br>- traffic volume (0)<br>- residential density (0)<br>- safety walking home night (0)	*no current PA; occasional PA; frequent PA (**in general**, not necessarily near home residence!)<br>**Litter, graffiti and lack of greenery (= absence of vegetation voluntarily displayed on outside walls, balconies, or windows). High score =2 or 3 /3 conditions ; moderate score = 1/3; low score = 0/3<br>*** low (vs high) physical disorder was associated with sign increase in

						**SAFETY**<br>- safety walking home at night	*WOMEN*:<br>- neighborhood disorder **(+)*****<br>- traffic volume(0)<br>- residential density (0)<br>- safety walking home night (0)	risk of occasional (vs no) sports, but not with “frequent vs no sports”
**Milosevic et al. 2009**<br><br>**Ref n° 56**	- n = 9,070<br>- age range: adults	Multistage stratified sampling	6 regions* in Croatia<br><br>Data collection 2003	CS	*Subjective:*<br>**TOTAL PA**<br>- PA	*Objective*<br>**URBANIZATION**<br>- “urbanization” (city Zagreb vs mountains=rural)	*Unknown analysis*<br>Most inactivity in city compared to mountains (least inactivity prevalence)<br>For both men and women	this paper discusses physical **Inactivity!**<br>* regions are [1] Eastern, [2] Northern, [3] Central, [4] City of Zagreb, [5] Mountainous and [6] Coastal
**Molina-García et al. 2010**<br><br>**Ref n° 57**	- n=518<br>- *M* age= 22.4y ± 5.3<br>- 59.7% F	Universities: Purposeful<br>Individuals: Convenience	2 universities in Valencia, Spain<br><br>Data collection April-May 2009	CS	*Subjective*:<br>**TOTAL PA**<br>**ACTIVE TRANSPORT**<br>- weekly energy expenditure ACU*<br>- total PA	*Objective:*<br>**ACCESS SERVICES**<br>- distance to university<br>*Subjective*:<br>**ACCES PUBLIC TRANSP**<br>- access to public transport (walking minutes)<br>**WALKING/CYCLING FACIL**<br>- presence and quality of walking and cycling facilities	*Correlations*<br>ACU<br>- distance to university (0)<br>- access to public transport (0)<br>- walking & cycling facilities **(+)**<br><br>TOTAL PA<br>- distance to university (0)<br>- access to public transport (0)<br>- walking & cycling facilities **(+)**	- ACU= active commuting to university. Participants could choose between bicycle, bus, car, metro/tram/tram, motorbike or walking<br>- walking and cycling facilities were assessed by 5 items of the NEWS questionnaire
**Ogilvie et al. 2008**<br><br>**Ref n° 58**	- n = 833 (AT)<br>- n = 684 (PA)<br>- *M* age 48y<br>- 61% F	Clusters: purposeful<br>Individuals: random<br><br>- r.r. = 15.9%	Three study areas (all extended from inner mixed-use districts close to the city centre to residential suburbs)in Glasgow, UK<br><br>Data collection	CS	*Subjective*<br>(IPAQ short version)<br>**ACTIVE TRANSPORT**<br>- active travel<br>**TOTAL PA**<br>- overall PA (meeting PHR)	*Objective*<br>**ACCESS SERVICES**<br>- travel distance to work (≥ 4 miles vs <4)<br><br>*Subjective*<br>(original scale items between brackets)<br>**AESTH**<br>- *aesthetics* (pleasant to walk; surroundings are unattractive)<br>**ACCESS RECR FACIL** - *green space* (park within walking distance; little green space)<br>**ACCESS SERVICES**<br>- *access to amenities*	*Multivariate logistic regression (personal and environmental model → adj for personal var^s^)*<br><br>- travel distance to work <4 miles **(+)**[1] (0)[2]<br>- aesthetics (0)[1,2]<br>- green space (0)[1,2]<br>- proximity to shops **(+)**[1] (0)[2]<br>- convenience of routes (0)[1,2]<br>- traffic volume **(+)**[2] (0)[1]<br>- road safety for cyclists **(-)**[1] (0)[2]<br>- personal safety (0)[1,2]<br>- travel distance to work **(-)**[1] (0)[2]	*Newly developed neighborhood scale*: 14 items; test-retest reliability: Ogilvie et al., 2008<br>- “active travel”= ≥30/d of walking/cycling/both<br>- objective environmental characteristics: concentric buffers from 100 to 500m around routes and access points of existing and planned motorways and around the network of other major roads. Each respondent was then assigned (based upon centroid of the residential postcode) to a category of proximity to each type of road infrastructure (within 100m; 101-200m etc.)

						(convenient public transport; nearest shop too far to walk)<br>**WALKING CYCLING FACIL**<br>- *convenience of routes* (convenient routes for walking; no convenient routes for cycling)<br>**TRAFFIC SAFETY**<br>- *traffic* (little traffic, lot of traffic noise)<br>- *road safety* (safe to cross the road; roads are dangerous for cycling)<br>**CRIME SAFETY** - *personal safety* (safe to walk after dark; people are likely to be attacked)		- for logistic regression analysis, the method of Hosmer and Lemeshow was used (first personal variables, then environmental)<br>- for the analyses, ≥*4miles* was set as the reference category<br>- **RESULTS**:<br>()[1] specific results for active travel<br>()[2] specific results for PA
**Owen et al. 2010**<br><br>**Ref n° 59**	- n = 372<br>- *M* age:<br>- MEN: 46.9y±15.7<br>- WOMEN: 44.8y ± 12.1<br>- 50.8% F	Clusters (Ghent): purposeful<br>Individuals: Convenience sampling	Ghent (BEL)<br><br>Data collection 2003	CS	*Subjective*<br>(IPAQ long form)<br>**CYCLING TRANSPORT**<br>- Bicycle use for transport*[1]	*Subjective*<br>(NEWS)<br>**WALKABILITY**<br>- neighborhood*[2] walkability	*Logistic regression*<br>*(adj for A, G, E and working status)*<br><br>- walkability **(+)**	*[1]classification into two categories: bicycle use for transport at least once a week vs less<br>*[2] "neighborhood" was defined as the local geographical area, 10 – 15 min walk around participant's home. Walkability was subdivided into 4 quartiles: low – high – higher – highest
**Panter et al. 2008**<br><br>**Ref n° 60**	- n = 401<br>- *M* age = 51.46 y ± 17.28<br>- 65.8% F	Clusters: purposeful<br>Individuals: Random<br><br>- r.r. = 45%	6 urban neighborhoods in the city of Norwich, South-East England, UK<br><br>Data collection: Aug and Sept 2004	CS	*Subjective*:<br>(EPAQ2)<br>**LTPA**<br>- sessions of aerobic PA<br>- sessions of all PA activities	*Objective:*<br>(ArcGIS)<br>**ACCESS RECR FACIL**<br>- accessibility of sports facilities<br>- accessibility of gyms	*Kruskal-Wallis tests:*<br>*(unadjusted!)*<br>Aerobic PA # sessions<br>- accessibility all facilities (0)<br>- accessibility sports facilities **(+)**<br>- accessibility gyms **(-)**<br>Overall PA sessions<br>- accessibility all facilities (0)<br>- accessibility sports facilities **(+)**<br>- accessibility gyms **(-)**	- neighborhoods were chosen to be of varying socio-economic deprivation: 2 more deprived, 2 middle ranking, 2 less deprived<br>- aerobic exercises were swimming, cycling, aerobics, visiting a gym, running or jogging, racket sports, football, netball or volleyball, cricket and martial arts. Other activities were walking, golf and light housework or gardening.<br>- sports facilities were defined as places that can be used to participate in a range of indoor or outdoor sports, or which had specialized

								equipment for one sport. Gyms were facilities which had only an indoor gymnasium available containing cardiovascular and/or weight training equipment.<br>- accessibility is considered in terms of <u>road distance</u> to facilities
**Panter & Jones 2008**<br><br>**Ref n° 61**	- n= 401<br>- mean age = 51.46 y ± 17.28<br>- 65.8% F	Clusters: purposeful<br>Individuals: Random<br>- r.r. = 45.2%	6 urban neighborhoods in the city of Norwich, South-East England, UK<br><br>Data collection: July 2005	CS	*Subjective:*<br>(EPAQ2)<br>**TOTAL PA**<br>- all PA ≥ 5 sessions/wk<br>**TOTAL WALKING**<br>- walking ≥ 5 sessions/wk<br>**LTPA**<br>- aerobic PA ≥ 5 sessions/wk	*Objective:*<br>(GPS and GIS)<br>**ACCESS RECR FACIL**<br>- accessibility *(= distance to)* sports facilities<br>- accessibility gyms<br>- accessibility parks and green spaces within which exercise can be undertaken<br><br>*Subjective:*<br>(NEWS)<br>**WALKABILITY**<br>- mean neighborhood walkability score<br>**GENERAL QUALITY**<br>- general neighborhood rating*[1]	*<u>Unknown analysis:</u>*<br>- accessibility parks/green (0)[1,2,3]<br>- accessibility gyms (0)[1,2,3]<br>- accessibility sports facilities **(+)**[1,2] (0)[3]<br>- walkability score **(+)**[1,2] (0)[3]<br><br>*<u>Logistic regression:</u>*<br>*(adj for A, G, E, income,* ***owning a dog****, *disliking exercise!)*<br><br>- *general neighborhood rating:* **(+)**[1] (0)[2,3]<br>- *accessibility park*:<br>**(+)**[1]<br>- *accessibility sports centre:* (0)[1,2,3]<br>- *accessibility gym:* (0)[1,2,3]	- neighborhoods were chosen to be of varying socio-economic deprivation: 2 more deprived, 2 middle ranking, 2 less deprived<br>- aerobic exercises were swimming, cycling, aerobics, visiting a gym, running or jogging, racket sports, football, netball or volleyball, cricket and martial arts.<br>Other activities were walking, golf and light housework or gardening.<br>- *<u>sports facilities</u>* were defined as places that can be used to participate in a range of indoor or outdoor sports, or which had specialized equipment for one sport. *<u>Gyms</u>* were facilities which had only an indoor gymnasium available containing cardiovascular and/or weight training equipment.<br>*[1] general neighborhood rating is a composite score, produced from 16 items adapted from the NEWS. It includes residential density, street connectivity, walking/cycling facilities, aesthetics and pedestrian traffic safety. Composite scores were then classed into tertiles for analyses<br>- "neighborhood" = area 10-15min around the participants residence<br>**RESULTS**<br>()[1]: specific results for general PA<br>()[2]: specific results for aerobic PA<br>()[3]: specific results for walking

								* Only adj for dog ownership in analyses for overall PA and walking! For the analyses, “poor” neighborhood rating, and furthest distances to facilities were set as the reference categories
**Panter et al. 2011**<br><br>**Ref n° 62**	- n = 1,279<br>- age range 49-80y<br>- mean age = 60.4y ± 5.4<br>- 61.1% F	Clusters: purposeful<br>Individuals: through GPs	EPIC-Norfolk Study, UK	CS	*Subjective*:<br>(EPAQ2)<br>ACTIVE TRANSPORT<br>- active commuting last year	*Objective:*<br>(GIS)<br>Objective environment<br>LAND USE MIX DIV<br>- land use mix<br>CONNECTIVITY<br>- junction density<br>ACCESS SERVICES<br>- distance to work<br>ACCESS RECR FACIL<br>- park in neighborhood<br>WALKING/CYCLING FACIL<br>- pavement density<br>TRAFFIC SAFETY<br>- density road traffic accidents<br>- density fatal and serious RTA<br>CRIME SAFETY<br>- crime rate<br>URBANIZATION<br>- urban-rural status<br>- building density<br><br>Objective route environment*[1]<br>LAND USE MIX<br>- land use mix score<br>CONNECTIVITY<br>- route length ratio*[2]<br>TRAFFIC SAFETY<br>- main road on route<br>- sec road on route<br>- main or sec road on route<br>- density of TRA on route<br>- density of fatal and serious	*X^2/ independent samples t-tests*<br>*Objective environment*<br>- land use mix **(-)**<br>- junction density **(+)**<br>- distance to work **(+)**<br>- park in neighborhood (0)<br>- pavement density **(+)**<br>- crime rate (0)^{m} **(+)**^{f}<br>- density RTA **(+)**<br>- density fatal and serious RTA **(+)**<br>- urban-rural status **(-)**<br>- building density **(+)**<br><br>*Objective route environment*[1]*<br>-land use mix score (0)^{m} **(+)**^{f}<br>- route length ratio** **(+)**<br>- main road on route **(-)**<br>- sec road on route **(-)**<br>- main or sec road on route**(-)**<br>- density of TRA on route **(+)**^{m} (0)^{f}<br>- density of fatal and serious RTA on route **(+)**^{m} (0)^{f}<br><br>*Subjective environment*<br>- land use mix diversity **(+)**<br>- street connectivity **(+)**<br>- access to services **(+)**<br>- walking and cycling facilities **(+)**<br>- pedestrian and traffic safety (0)^{m} **(+)**^{f}<br>- safety from crime **(+)**^{m} (0)^{f}	- participants had to be living within a 10 km distance from work location<br>- active transportation question had the following answer categories: always, usually, occasionally, never/rarely. Active commuters were those who reported “always” or “usually” traveling to work by bicycle or on foot.<br>- neighborhoods were defined as the area within an approximate 10-min walk (~800m) of participants’ postcodes<br>*[1] shortest routes between home and work locations were calculated, and seven measures representing environmental characteristic of the zone within 100m surrounding it were estimated<br>*[2] route length ratio = road length/ straight distance<br>- a combined best-fit model was used to investigate the potential mediating effects of psychological factors on the relationship between distance, environmental predictors, and AT<br>- **RESULTS**<br>(0)^{m} = specific results for men<br>(0)^{f} = specific results for women<br>()= results are the same for men& women

						RTA on route<br><br>*Subjective:*<br>(NEWS)<br>**LAND USE MIX**<br>- land use mix diversity<br>**CONNECTIVITY**<br>- street connectivity<br>**ACCESS SERVICES**<br>- access to services<br>**WALKING/CYCLING FACIL** -<br>walking and cycling facilities<br>**TRAFFIC SAFETY**<br>- pedestrian and traffic safety<br>**CRIME SAFETY**<br>- safety from crime<br>**AESTHETICS**<br>- aesthetics	- aesthetics (0)	
**Parkes & Kearns 2006**<br><br>**Ref n° 63**	- n = 14,602<br>- age range = 16y – 59y<br>- 57.7%F	Clustering? Systematic random sampling, including the 4 main Scottish cities<br><br>Clusters: purposeful<br>Individuals: random	Scotland, UK<br><br>Scottish Household Survey 2001	CS	*Subjective*:<br>**WALKING TRANSPORT**<br>- walking to get somewhere ≥ 5 d/wk<br>**WALKING RECR**<br>- walking for fitness/ pleasure/ exercise the dog ≥ 4 d/wk	*Subjective:*<br>**ACCESS SERVICES**<br>- convenience local facilities<br>**SAFETY**<br>- safety neighborhood to walk in evening<br>**CRIME SAFETY**<br>- experienced crime<br>**AESTHETICS**<br>- neighborhood vandalism/litter<br>- like neighborhood appearance<br>- like neighborhood peace and quiet	<u>*Logistic regression*</u><br>*(adj for A, G, social tenure, household access to a motor vehicle, smoking, disability)*<br>- convenience local facilities **(+)**[1] ⁽0)[2]<br>- experienced crime (0)[1,2]<br>- safety neighb to walk in evening **(-)**[2] (0)[1]<br>- neighb vandalism/litter **(+)**[1] (0)[2]<br>- like neighb appearance **(+)**[1,2]<br>- like neighb peace and quiet (0)[1,2]	- walking was assessed by asking the # d/wk people undertook trips of > a quarter of a mile.<br>**-** in the walking for fitness group, running and jogging were included!<br>- for the analyses, the reference categories were “no experienced crime”; “safe to walk in evening”; “lowest tertile vandalism/litter”; “not liking neighborhood’s appearance”; “not liking neighborhood’s peace and quiet”;highest tertile of convenience of services<br>**RESULTS**:<br>()[1]: specific results commuting<br>()[2]: specific results fitness/pleasure
**Parkin et al. 2008**<br><br>**Ref n° 64**	- n = 8,800 electorial wards<br>- age range 16y – 74y<br>- 45% F	Census data 2001	England and Wales, UK	CS	*Objective:*<br>**CYCLING TRANSPORT**<br>- proportion cycling to work*[1] of the 8,800 wards in England and	*Objective* :<br>**ACCESS SERVICES**<br>- distance travelled to work<br>**WALKING:CYCLING FACIL**<br>- prop. principal road length	<u>*Logistic regression:*</u><br>- *distance* travelled to work 2-5km **(-)**; 5-20km **(-)**; other (0)→ **(-)**<br>- prop. principal road length	*[1] the term “cycling to work” also includes bicycle journeys for education for those aged 16 and over<br>- distance travelled to work is calculated based on a straight line

					Wales	deemed to have failed (~<u>bad</u> quality)<br>- prop. non-principal road length deemed to have failed (~ <u>bad</u> quality)<br>- prop. road and cycle route that is signed (on a map)<br>- prop. cycle route that is off-road<br>- prop. cycle route that is adjacent to the road<br>- prop. road that has a bicycle or bus lane<br>**TRAFFIC SAFETY**<br>- transport demand intensity<br>**URBANIZATION**<br>- population density<br>**HILLINESS**<br>- prop. 1 km² ≥3% mean slope (and ≥4%)<br><br>*Subjective*:<br>**TRAFFIC SAFETY**<br>- prop. probability of acceptability of cycling (risk perception in different cycling circumstances)	deemed to have failed **(-)**<br>- prop. non-principal road length deemed to have failed **(-)**<br>- prop. road and cycle route that is signed (0)<br>- prop. cycle route off-road **(+)**<br>- prop. cycle route adjacent to the road (0)<br>- prop. road with bicycle/bus lane (0)<br>- transport demand intensity **(-)**<br>- population density **(+)**<br>- prop. 1 km² ≥3% mean slope (and ≥4%) **(-)**<br>- prop. probability of acceptability of cycling (0)	between centroids of residence and workplace postcodes: [1]<2km; [2]2-5km; [3]5-10km; [4]10-20km; [5]20-30km; [6]30-40km; [7]40-60km; [8] ≥60km; all at ward level! For the analyses, distance <2km, was taken as reference category<br>- population density serves as a proxy for **urbanization** degree of a district<br>- measure for hilliness relates to general topography of a district (not spec to hilliness of routes within the district)<br>- no correlations between population density and hilliness (➔ no different effects in **rural and urban areas**)<br>- transport demand intensity serves as a proxy for the condition of the infrastructure for cycling<br>- 81.6% of the variation in cycling to work is explained by the model<br>*No significant interactions between hilliness and [1] distance, [2] highway condition, [3] transport demand intensity, [4] population density and [5] provision of off-road route*
**Pascual et al. 2009**<br><br>**Ref n° 65**	- n= 25,982<br>- *M* age= 49.4y<br>- 51.3%F	Clusters (provinces):<br>Individuals: Random multi-stage stratified procedure<br><br>- r.r.= 70%	Spain<br>One sample for each of Spain's 50 provinces<br><br>Data collection 1999	CS	*Subjective:*<br>- swimming last 30 d (y/n)<br>- gym use last 30 d (y/n)	*Objective*:<br>- # swimming pools / 10,000 population<br>- # gyms / 10,000 population	<u>*Random effect logit models with random intercept**</u><br><u>Swimming</u><br>- # swimming pools/10,000 population (0)<br>(same results 25-49y & 50-74y)<br><br><u>Gym use</u><br>- # gyms/10,000 population (0)<br>(same results 25-49y & 50-74y)	Data about PA from the 1999 general survey on customs regarding media and leisure activities<br>- facilities for the practice of PA were used as an indicator of the availability of gyms. Other facilities for team sports, or for other individual sports , were excluded<br>- n° of swimming pools and gyms were estimated in each province<br>- also subdivision in 25-49y and 50-74y; then there were some differences in the relationship PA and

								socioeconomic environment<br>* clusters were based on province
**Pitsavos et al. 2005**<br><br>**Ref n° 66**	- n = 3,042<br>MEN<br>- *M* age= 46y± 13<br>WOMEN<br>- *M* age= 45y± 13<br><br>- 50% F	Multistage random sampling<br><br>- r.r. = 75%	Attica region, urban and rural areas, Greece<br>ATTICA study<br><br>Data collection May 2001 – August 2002	CS	*Subjective:*<br>**LTPA**<br>- LTPA (light, mod, high)	*Objective:*<br>**URBANIZATION**<br>- urbanization (urban vs rural)	*X^2 test*<br>- urbanization **(-)**<br>(urban 46%, rural 55%, p=0.02)	- LTPA was assessed by a validated questionnaire concerning weekly energy expenditure and categorized as “low” (1st tertile); “medium” (2nd tertile) or “high” (3rd tertile)
**Poortinga 2006**<br><br>**Ref n° 67**	- n = 14,836<br>- *M* age= 48.21y±18.49<br>- 55.5% F	Clusters: purposeful<br>Individuals: random	Health Survey for England (UK)<br><br>Data collection June 2003 – March 2004	CS	*Subjective:*<br>**TOTAL PA**<br>overall PA: active ≥ 5 d/wk ≥30 min*[1]<br>**LTPA**<br>- sports activity: ≥ 2 d/wk ≥ 30min<br>**TOTAL WALKING**-<br>- walking (all): ≥ 1d/wk ≥ 30 min	*Subjective:*<br>**ACCESS SERVICES**<br>- easiness to get to supermarket<br>- easiness to get to post office<br>**ACCESS RECR FACIL**<br>- good leisure things for people like myself in neighborhood<br>**CRIME SAFETY**<br>- teenagers hanging out<br>- problems of vandalism, graffiti or deliberate damage to property<br>**URBANIZATION**<br>*Urbanization* (urban, suburban, rural)	*Multilevel analysis (3-level logistic regression)*<br>- *access to amenities:*<br>- good leisure things **(+)**[1,2] (0)[3]<br>- access supermarket (0)[1,2,3]<br>- access post office **(+)**[1,3] (0)[2]<br>- *social nuisances:*<br>- teenagers hanging out (0)[1,2,3]<br>- problems vandalism, graffiti, deliberate damage **(+)**[1] (0)[2,3]<br>- *urbanization:* (0)[1,2,3]<br>[- suburb **(-)**[3] (0)[1,2]<br>- rural (0)[1,2,3]]	*[1] activities included all PA, such as housework, home-based manual work, walking, occupational activity and sports in the last 4 weeks<br>- access to amenities was assessed by asking how easy it was to reach places using the “usual type of transport”!<br>- for the analyses, “urban” was set as the reference category<br>**RESULTS:**<br>()[1]: specific results for overall PA<br>()[2]: specific results for sports<br>()[3]: specific results for walking
**Rütten et al. 2001**<br><br>**Ref n° 68**	*Total group*<br>- n = 3,343<br>- *M* age = 47y ±16.92<br>- 56.9% F	Random<br>- overall r.r. = 53.5%	Six European countries: Belgium, Finland, Germany (E+W), The Netherlands, Spain, Switzerland<br><br>MAREPS project	CS	*Subjective:*<br>**TOTAL PA**<br>- PA	*Subjective:*<br>**ACCESS RECR FACIL**<br>- opportunities for PA	**TOTAL GROUP**<br>*Zero order correlation analysis: (Pearson coefficients)*<br>- opportunities for PA **(+)**<br>*ANOVA*<br>Active groups vs “inactive”<br>- opportunities for PA **(+)**[1,2,3]<br>*MEN*<br>Active groups vs “inactive”<br>- opportunities for PA **(+)**[1,2,3]<br>Active groups vs one another<br>- light/mod more opportunities	- item concerning PA: do you do any gymnastics, PA or sports? (y/n) + if yes, vigorousness was assessed.<br>- the two items were combined to a 6 point scale, so that “0”= no PA; “1” to “5”= different levels of vigorousness<br>- “opportunities for PA” = composite score of 3 items (residential area, local service providers and community itself)<br>- “inactive”: vigorousness = 0;<br>**RESULTS:**

							than vigorously<br>- light/mod more opportunities than very vigorously<br>- no differences between vigorously and very vigorously<br>*WOMEN*<br>Active groups vs "inactive"<br>- opportunities for PA **(+)**[1,2,3]<br>Active groups vs one another<br>No differences between active groups	()[1]:specific results for "light/mod active" : (vig.= 1,2,3)<br>()[2]: specific results for "vigorously active": (vig. = 4)<br>()[3]: specific results for "very vigorously active":(vig. = 5)
**Rütten & Abu-Omar 2004**<br><br>**Ref n° 69**	- n = 16,230<br>- 53.7% F	Multi-stage, random sample design<br><br>- mean r.r. across all countries: 54.6%<br>(range 23%-84%)	Fifteen European countries: Belgium, Denmark, Eastern Germany, Western Germany, Greece, Spain, France, Ireland, Italy, Luxembourg, The Netherlands, Austria, Portugal, Finland, Sweden, Great Britain and Northern Ireland (MAREPS project)	CS	*Subjective:*<br>(face-to-face interviews; IPAQ short version last 7d)<br>**TOTAL PA**<br>- total PA (MET-h/wk)	*Subjective:*<br>**ACCESS RECR FACIL**<br>- many opportunities for PA offered in the area where participants live	*Spearman's correlations:*<br>Countries:<br>Austria **(+)**<br>Belgium **(+)**<br>Denmark **(+)**<br>Finland **(+)**<br>France**(+)**<br>Germany (West) **(+)**<br>Germany (East) **(+)**<br>Great Britain (0)<br>Greece **(+)**<br>Ireland **(+)**<br>Italy **(+)**<br>Luxembourg **(+)**<br>Netherlands (0)<br>Northern Ireland **(+)**<br>Portugal **(+)**<br>Spain **(+)**<br>Sweden (0)	- "opportunities for PA" = composite score of 3 items (residential area, local service providers and community itself); test-retest reliability after 4 to 7 days (Spearman's Rho of 0.65 – 0.71 for the 3 items)<br>- PA in MET-hours/wk, divided into 4 quartiles: [1] ≤ 8; [2] 8.01 – 24; [3] 24.01 – 51.1; [4] ≥ 51.11
**Santana et al. 2009**<br><br>**Ref n° 70**	- n = 7,669<br>- age range ≥18y (78% <65y)<br>- 53.5% F	Clusters: purposeful<br>Individuals: unknown sampling method	143 neighborhoods in Lisbon metropolitan area (LMA), Portugal<br><br>Data collection 1998-1999	CS	*Subjective:*<br>**LTPA**<br>- planned moderate PA ("/walking" in table)<br>- planned vigorous PA	*Objective:*<br>**ACCESS SERVICES**<br>- n° of groceries<br>- n° of supermarkets<br>- n° of post offices<br>- n° of public health services<br>**ACCESS RECR FACIL**<br>- n° of green parks<br>- n° of sports facilities	*Multilevel (2) logistic regression (adj for demographic, economic activity, E, income and behavioral variables)*<br>Moderate PA+ walking<br>- n° of post offices (0)<br>- n° of public health services **(+)**<br>- n° of green parks (0)<br>- n° of swimming pools (0)	

						**TRAFFIC SAFETY**<br>- traffic accidents<br>**CRIME SAFETY**<br>- crime<br>**URBANIZATION**<br>- urban sprawl (pop density)	- traffic accidents with victims (0)<br>- crimes against property **(-)**<br>- population density (0)<br>Vigorous PA<br>- n° of gymnasiums **(+)**<br>- n° of green parks (0)<br>- n° of swimming pools **(+)**<br>- traffic accidents with victims (0)<br>- crimes against property (0)<br>- population density (0)	
**Santos et al. 2009**<br><br>**Ref n° 71**	- n = 7,330<br>- age range 18-65y<br>- *M* age = 38.1y ± 9.3y<br>- 56.0% F	Cluster (islands): purposeful<br>Individuals: all parents of school children<br><br>- r.r. = 87.6%	Azorean PA and Health Study (APAHS); Azorean Archipelago, Portugal, North Atlantic<br><br>Data collection 2004	CS	*Subjective:*<br>(IPAQ short form)<br>**TOTAL WALKING**<br>- walking (min/wk)*	*Subjective:*<br>(environmental module of the IPS):<br>Dimension1:<br>**GENERAL QUALITY**<br>Infrastructures, access to destinations, social environment and aesthetics<br><br>Dimension2:<br>**SAFETY**<br>Neighborhood safety	*Chi² tests (unknown)*<br>Walking:<br>- dimension 1 **(-)**<br>- safety **(+)**<br><br>*Multilevel (adjusted for A, G, E??) regression*<br>Walking:<br>- dimension 1 **(+)**<br>- safety (0)	* walking was classified as *never* (0min previous wk); *occasional* (≥10min/wk, but < 150min/wk); or *regular* (≥150min/wk)<br>- 15 environmental items in survey were assessed on the basis of 4-point likert scales, 1 open-ended item and 1 item with 6 response categories<br>- two environmental dimensions were assessed after CATPCA (categorical principal components analysis) with acceptable/good reliability (Cronbach's Alpha >0.6)<br>- for regression analysis, the indexes of the dimensions were used as continuous variables<br>- 3 levels for multilevel regression: level1 – subject; level2 – municipali-ty; level3 – island of residence<br>- perceptions environment: pos vs neg
**Santos et al. 2008**<br><br>**Ref n° 72**	- n = 7,330<br>- age range 18-65y<br>- *M* age = 38.1y ± 9.3y<br>- 56.0 % F	Clusters: purposeful<br>Individuals: random (?)<br><br>- r.r. = 87.6%	Azorean PA and Health Study (APAHS)<br>Portugal<br>Data collection 2004	CS	*Subjective:*<br>(IPAQ short form)<br>**TOTAL PA**<br>- moderate PA<br>- HEPA	*Subjective:*<br>(environmental module of the IPS)<br>Dimension1:<br>**GENERAL QUALITY**<br>Infrastructures, access to	*Multilevel logistic regression (adj for A, BMI, E and island of residence)*<br><br>*MEN*<br>Moderate PA (0)	* Low PA (not meeting criteria for moderate PA or HEPA)<br>Moderate PA (≥3d vigorous PA for ≥20'; ≥5d mod PA/walking ≥30'; ≥5d any combination walking/mod PA/vig PA achieving ≥600MET-min/wk)

						destinations, social environment and aesthetics<br><br>Dimension2:<br>**SAFETY**<br>Neighborhood safety	HEPA (0)<br><br>*WOMEN*<br>Moderate PA<br>- dimension 1 **(+)**<br>- dimension 2 (0)<br>HEPA<br>- dimension 1 **(+)**<br>- dimension 2 (0)	HEPA (vig PA ≥3d/wk achieving ≥1500MET-min/wk; ≥5d/wk any combination walking/mod PA/ vig PA achieving ≥3000 MET-min/wk)<br>- level1 – subject; level2 – municipality; level3 – island of residence<br>- perceptions environment: reference = negative perception
**Shenassa et al. 2006**<br><br>**Ref n° 73**	- n = 5,338<br>- *M* age = 40.7y ± 13.6<br>- 53.9% F	Clusters (cities): purposeful<br>Individuals: random	Eight European cities: Angers (FR), Bonn (GER), Bratislava (SVK), Budapest (HU), Ferreira do Alentejo (POR), Forli (ITA), Geneva (SWI), Vilnius (LIT)<br>- data collection LARES project, WHO 2002-2003	CS	*Subjective:*<br>**LTPA**<br>- occasional exercise<br>- frequent exercise (mod or vig intensity)<br><br>→ Exercise in general*[1]	*Subjective:*<br>**ACCESS RECR FACIL**<br>- access to green space<br>**SAFETY**<br>- safety*[1]<br>**AESTH**<br>- litter on street	*Logistic regression analysis (adj for A, G, E, marital status, disability status, family size, city of residence*<br>*MEN AND WOMEN*<br>Occasional exercise<br>- unsafety **(+)**[1] (0)[2]<br>- litter **(-)**[1,2]<br>- access green space (0)[1,2]<br><br>MEN<br>Occasional exercise<br>- unsafety **(+)**[1] (0)[2]<br>- litter **(-)**[2] (0)[1]<br>- access green space (0)[1,2]<br><br>WOMEN<br>Occasional exercise<br>- unsafety **(+)**[1,2]<br>- litter (0)[1,2]<br>- access green space (0)[1,2]	*[1] "do you feel safe returning to your home when it is dark?"<br>- for the analyses, "safe" and "no access to green space" were set as the reference categories<br>**RESULTS**:<br>()[1]: specific results for occasional exercise<br>()[2]: specific results for frequent exercise
**Sigmundová et al. 2011**<br><br>**Ref n° 74**	- n = 649<br>- *M* age = 36.29y ± 13.04<br>- age range = 18y – 69y<br>- 57.9% F	Clusters (towns): purposeful<br>Individuals: random	Eight regional towns (>90,000 inhabitants) in the Czech Republic<br>Data collection 2007	CS	*Objective*:<br>(Yamax digiwalker SW-700 pedometer)<br>**TOTAL PA**<br>- Achieving health enhancing guidelines: 10,000 steps/d	*Subjective*:<br>(ANEWS)<br>**ACCESS SERVICES**<br>- accessibilityshops/non-sports facilities<br>- accessibility of services in a neighborhood	*Spearman correlation analysis*<br>(corr with daily step count)<br>- pleasantness environment **(+)**[1,2]<br>- safety (0)[1,2]<br>- walking-friendliness (0)[1,2]<br>- better types of residences in	- for the logistic regression analyses, "unpleasant environment", "better accessibility of shops", and "less safe neighborhood" were set as reference categories<br><br>**- RESULTS:**

						**ACCESS RECR FACIL**<br>- distance to sports facilities<br>**WALKING/CYCLING FACIL**<br>- walking-friendly environment<br>- locations for walking and cycling<br>**SAFETY**<br>- safety<br>**AESTH**<br>- better types of residences in neighborhood<br>**GENERAL QUALITY**<br>- pleasantness environment	neighborhood **(+)**[1] (0)[2]<br>- accessibility shops/non sports facilities in minutes **(-)**[2] (0)[1]<br>- distance sports facilities (0)[1,2]<br>- accessibility services (0)[1,2]<br>- walking/cycling locations (0)[1,2]<br><br>*Logistic regression analysis*<br>(achieving 10,000 steps/d)<br>*7 DAYS*<br>- pleasantness environment **(+)**<br>- safety (0)<br>- accessibility shops **(-)**<br>- accessibility sports facilities (0)<br><br>*WORKING DAYS (5 DAYS)*<br>- pleasantness environment **(+)**<br>- safety **(+)**<br>- accessibility shops **(-)**<br>- distance sports facilities (0)	()[1]: specific results for men<br>()[2]: specific results for women
**Stafford et al. 2007**<br><br>**Ref n° 75**	- n = 7,023<br>- *M* age = 38.6y<br>- 54% F	Clusters: purposeful<br>Individuals: random	438 postcode sectors in UK: London, England, Scotland (health survey England and Scotland)<br>Data collection 1994-1999	CS	*Objective:*<br>**LTPA**<br>- av proportion of people participating in sports clubs	*Subjective:*<br>**CRIME SAFETY**<br>- neighborhood disorder	*Structural equation modeling*<br>- neighborhood disorder **(-)**	- postcode sectors have and av population of 5,000<br>- neighborhood disorder was determined by n° of special constables, n° of police officers, vacant/derelict land (and non sign by violent crime rate and missed waste collections)
**Stahl et al. 2001**<br><br>**Ref n° 76**	- n total = 3,343<br>- *M* age = 47y ± 16.9<br>- 56.9% F	Clusters: purposeful<br>Individuals: random (exception = NL)<br>- overall r.r. = 53.5%	MAREPS project Belgium, Finland, Germany, The Netherlands, Spain, Switzerland<br><br>Data collection 1997 -1998	CS	*Subjective:*<br>**TOTAL PA**<br>- PA (active vs inactive)	*Subjective:*<br>**ACCESS RECR FACIL**<br>- local opportunities for PA	*Bivariate analyses:*<br>- high local opportunities for PA **(+)**	- "Local opportunity" is a scale covering 3 items, (Cronbach's α = 0.74) and were categorized as low or high using median as cut off point. Items were "my residential area offers many opportunities to be PA" ; "local sports clubs and other providers in my community offer many opportunities" and "my community doesn't do enough for the citizens and their PA"<br>- PA was assessed by the item "do

								you do any gymnastics, PA or sports"<br>**RESULTS**:<br>()[1] = specific results for statement "the area where I live offers me many opportunities to be PA"<br>()[2] = specific results for statement "local sport clubs and other providers offer many opportunities to be PA"<br>()[3] = specific results for statement "my local authority does enough for its citizens concerning their PA"
**Stronegger et al.<br>2010**<br><br>**Ref n° 77**	- n = 997<br>- age range = 15y-60y<br>*M* age = 37.61y±12.67<br>- 50.9% F	Individuals: random<br><br>- r.r. = 69.2%	Graz, Austria<br><br>Data collection October 2005	CS	*Subjective:*<br>**LTPA**<br>- LTPA(min/wk)*[1]<br>**WALKING TRANSPORT**<br>- walking for transportation<br>**CYCLING TRANPORT (2x)**<br>- cycling for transportation summer<br>- cycling for transportation winter	*Subjective:*<br>**ACCESS SERVICES**<br>- local infrastructure*[3]<br>**GENERAL QUALITY**<br>- general social-environmental quality*[2]	*multiple linear regression*<br>*(adj for A)*<br><br>*MEN*<br>LTPA:<br>- social-envir quality **(+)**<br>- local infrastructure (0)<br>Walking transportation:<br>- social-envir quality (0)<br>- local infrastructure (0)<br>Cycling transportation summer:<br>- social-envir quality (0)<br>- local infrastructure **(+)**<br>Cycling transportation winter:<br>- social-envir quality (0)<br>- local infrastructure **(+)**<br><br>*WOMEN*<br>LTPA:<br>- social-envir quality **(+)**<br>- local infrastructure (0)<br>Walking transportation:<br>- social-envir quality (0)<br>- local infrastructure **(+)**<br>Cycling transportation summer:<br>- social-envir quality (0)<br>- local infrastructure (0)<br>Cycling transportation winter:<br>- social-envir quality (0)<br>- local infrastructure (0)	*[1] at least one vigorous-intensity PA in the last 7 days<br>*[2] reputation/appearance of the quarter; location of the quarter within the city; safety within the quarter; possibilities of recreational walking; environmental quality such as quietness or air quality<br>*[3]connection public transport, infrastructure shops and medical services, accessibility of leisure time facilities and recreational resources

**Sundquist et al. 2011**<br><br>**Ref n° 78**	- n = 2,269<br>- age range = 18y – 65y<br>*M* age =45.8y<br>- 55% F	Clusters: purposeful<br>Individuals: random	32 highly and less walkable neighborhoods in Stockholm, Sweden<br>(SNAP)	CS	*Objective:*<br>(accelerometer 7d)<br>**TOTAL PA**<br>- MVPA<br><br>*Subjective:*<br>(IPAQ)<br>**WALKING TRANSPORT**<br>- walking for transport<br>**WALKING RECR**<br>- walking for leisure	*Objective:*<br>(GIS)<br>**WARLKABILITY**<br>- neighborhood walkability	*Multilevel linear regression*<br>*(crude analyses)*<br>MVPA<br>- walkability **(+)**<br>Walking for active transport<br>- walkability **(+)**<br>Walking for leisure<br>- walkability **(+)**<br><br>→ only crude analyses were used for summary calculations, similar outcomes for adjusted regression	- protocols similar to BEPAS, PLACE and NQLS<br>- subdivision into neighborhoods based on walkability and income (both high vs low)→ 4 types<br><br>SES-effect comparable to Belgium and US, with no neighborhood SES effects
**Titze et al. 2007**<br><br>**Ref n° 79**	- n = 538<br>- *M* age = 23.8y ± 3.5<br>- 43.9% F	Purposeful (students were recruited during university classes)<br><br>- r.r. = 71.1%	Austria, University students living in city of Graz<br><br>Data collection Spring 2004	CS	*Subjective:*<br>**CYCLING TRANSPORT**<br>cycling for transportation last 7 d*[1]<br>[None – irregular – regular]	*Objective:*<br>Cycle routes from home to university (GIS)<br><br>*Subjective:*<br>**TRAFFIC SAFETY**<br>- traffic safety on way to uni<br>**CRIME SAFETY**<br>- safety from bicycle theft<br>**AESTH**<br>- attractiveness route	*Multi-nominal regression analysis*:<br>*(adjusted for A, G, economic situation and distance from home to university and exercise level?)*<br><br>Irregular cyclists (1-3x/wk): (group 1)<br>- traffic safety on way to uni (0)<br>- attractiveness route **(+)**<br>- safety from bicycle theft **(+)¥**<br><br>Regular cyclists (>3x/wk): (group 2)<br>- traffic safety on way to uni **(-)**<br>- attractiveness route (0)<br>- safety from bicycle theft **(+)**	*[1] non cyclists (< 1d/7 to university); irregular (1-3x/7 to university); regular (≥ 3x/7 to university)<br>¥ trend towards significance, as p-value ranges between 0.05 and 0.10<br>– for the analyses, "non cyclists" were set as the "reference category"<br>**-** only subjective environmental measures were used for the summary calculations
**Titze et al. 2008**<br><br>**Ref n° 80**	- n = 905<br>- *M* age = 37.6±12.7<br>- 50.8% F	Individuals: random (random digit dialing method)<br><br>- r.r. = 69.3%	City of Graz, Austria<br><br>Data collection 2005	CS	*Subjective:*<br>**CYCLING TRANSPORT**<br>- cycling for transportation *	*Objective*:<br>- distance to destination (GIS)<br>→ *not used for summary calculations*<br><br>*Subjective*:	*Bivariate* X^2 *analyses*<br>*Unadjusted*<br>- land use mix diversity (0)<br>- bike lane connectivity **(+)**<br>- presence of sidewalks (0)<br>- presence streetlights night (0)	* cyclists were those who bicycled ≥1x/last 7d

						**LAND USE MIX**<br>- land use mix diversity of uses<br>**WALKING/CYCLING FACIL**<br>- bike lane connectivity<br>- presence of sidewalks<br>**SAFETY**<br>- presence of streetlights at night<br>**TRAFFIC SAFETY**<br>- safety from traffic<br>**AESTH**<br>- attractiveness of cycling conditions<br>**HILLINESS**<br>- presence of steep elevation	- safety from traffic (0)<br>- attractiveness of cycling conditions (0)<br>- presence of steep elevation **(+)**	
**Toftager et al. 2011**<br><br>**Ref n° 81**	- n = 11,092<br>- *M* age = 48.9y<br>- 53.7%F	Clusters: purposeful<br>Individuals: Random<br><br><br>- r.r. = 66.7%	Denmark<br><br>Data collection 2005	CS	*Subjective:*<br>(face to face interview)<br>**LTPA**<br>- using green space for exercise (self-administ)<br>- MVPA leisure time *[1](past year)	*Objective:*<br>**URBANIZATION**<br>- size of municipality*[2]<br>*Subjective:*<br>(self-administered questionnaire)<br>**ACCESS RECR FACIL**<br>- distance to green space	Multiple logistic regression<br>Use of green space for exercise<br>Municipality size: **(+)**<br>Distance to green space: **(-)**<br><br>MVPA leisure time<br>*(adj for A, G, combined school and vocational edu, accommodation type, size of municipality and long-term activity limitation)*<br>Distance to green space: **(-)**	*[1] MVPA in leisure time was categorized as "heavy exercise and competitive sports regularly and several times/wk" OR as "exercise or heavy gardening at least 4hours/wk<br>*[2] municipality sizes: [1]; <10,000inh; [2]10,000-<20,000inh; [3] 20,000-<40,000inh; [4]40,000-<100,000inh and [5]≥100,000inh<br>*[3] self-reported distance to different kinds of green space was divided into [1]<300m; [2]300m-1km; [3]1km to 5km; and [4]>5km; and was asked in the settings "beach", "sea", "lake", "park", "urban green space", "forest" and "open green space". These variables were grouped into 1 "green space" variable<br>- for the analyses, <300m distance to green space and municipality size <10,000inh were set as reference categories

**Van Dyck et al.**<br>**2009**<br><br>**Ref n° 82**	- n = 120<br>- age range = 20y – 65y<br>- *M* age = 43.0y ± 13.3<br>High walk: 56.7% F<br>Low walk: 51.7% F	Clusters: purposeful<br>Individuals: random<br><br>- r.r. = 47.8%	One high and one low walkable neighborhood in Sint-Niklaas, Belgium<br><br>Data collection Nov Dec 2006	CS	*Objective:*<br>**TOTAL PA**<br>- step count/d (Yamax digiwalker SW-200 pedometer; 7 days)<br><br>*Subjective*: AT<br>(adj. version of NPAQ)<br>**WALKING TRANSPORT**<br>- walking for transport in neighborhood (min)<br>**CYCLING TRANSPORT**<br>- cycling for transport in neighborhood (min)<br>**WALKING RECREATION**<br>- walking for recreation in neighborhood (min)<br>**CYCLING RECREATION** - cycling for recreation in neighborhood (min)	*Objective*:<br>**WALKABILITY**<br>Neighborhood walkability*	*<u>Independent samples t-tests:</u>*<br><br>- steps per day **(+)**<br><br>*In neighborhood:*<br>- walking *transport* **(+)**<br>- cycling transport (0)<br>- walking recreation (0)<br>- cycling recreation (0)<br><br>*Outside neighborhood:*<br><br>*<u>Two-way ANOVAs:</u>*<br><br><u>preference AT</u><br>- step count (0)<br>*<u>preference PT</u>*<br>- step count **(+)**	- AT= active transport<br>- PT = passive transport<br>* objective data gathered by field observation 800m radius, as GIS was not available for the city St-Niklaas<br>- only PA "in" neighborhood was taken up for the summary calculations
**Van Dyck et al.**<br>**2010**<br><br>**Ref n° 83**	- n = 1,166<br>- age range: 20-65y<br>- *M* age = 42.7y ±12.6<br>- 52.1% F	Clusters: purposeful<br>Individuals: random<br><br>- r.r. = 58.0%	BEPAS: 24 neighborhoods* in Ghent, Belgium<br><br>Data collection May 2007-September 2008	CS	*Objective*:<br>(CSA accelerometers, model 7164))<br>**TOTAL PA**<br>- MVPA (min)<br><br>*Subjective*:<br>(IPAQ, long form last 7d)<br>**WALKING TRANSPORT**<br>- walking for transport<br>**CYCLING TRANSPORT**<br>- cycling for transport<br>**WALKING RECR**<br>- recreational walking	*Objective:*<br>(GIS)<br>**WALKABILITY**<br>- neighborhood walkability	*<u>Multivariate multi-level analyses:</u>*<br>*(unadjusted)*<br>- CSA MVPA **(+)**<br>- walking for transport **(+)**<br>- cycling for transport **(+)**<br>- recreational walking **(+)**	* neighborhoods were stratified upon walkability and SES (4 different types)<br>- BEPAS = Belgian Environmental PA Study<br>- walkability was calculated based upon 3 elements: land use mix (5 types: residential, retail, office, institutional and recreational), intersection density and residential density<br>- logarithmic transformations (log10) were used to improve normality of the PA variables<br>- for the multi-level analyses, age and BMI were centered on the grand mean
**Van Dyck et al.**<br>**2011**	- n = 350<br>- age range: 20-65y	Clusters: purposeful<br>Individuals:	5 rural and 5 urban neighborhoods in	CS	*Objective:*<br>**TOTAL PA**<br>- step count/d (Yamax	*Objective*:<br>**URBANIZATION**<br>- urbanization*	*X^2 and independent sample t-tests*: no differences urban vs rural on sociodemographic	* urban vs rural (rural= reference cat)<br>- for every minute of cycling or swimming reported, 150 steps were

**Ref n° 84**	<u>Urban pp:</u><br>- *M* age = 41.7y ±13.5<br>- 37.9% M<br><u>Rural pp:</u><br>- *M* age = 43.1y ±12.8<br>- 41.1% M	random<br><br>- r.r. = 30.4%	Flanders, Belgium (Boeckhout-Vremde, Oordegem, Zaffelare, Ghent, Antwerp, Aalst)<br><br>Data collection: Oct 2008 – March 2009		digiwalker SW-200 pedometer; 7 days)<br><br>*Subjective*:<br>(adj version of NPAQ)<br>**WALKING TRANSPORT**<br>- walking for transport in neighborhood (min)<br>**CYCLING TRANSPORT**<br>- cycling for transport in neighborhood (min)<br>**WALKING RECR**<br>- walking for recreation in neighborhood (min)<br>**CYCLING RECR**<br>- cycling for recreation in neighborhood (min)<br>**LTPA**<br>- moderate LTPA (min)<br>- vigorous LTPA (min)		characteristics, except for educational level and working situation<br><br><u>*ANCOVA*</u> *(E = covariate)*<br><br>- mean step count weekdays **(+)**<br>- mean step count weekend days (0)<br>- mean step count whole week **(+)**¥<br>- moderate LTPA (0)<br>- vigorous LTPA (0)<br><br>*In neighborhood*:<br>- walking transport in neigh **(+)**<br>- cycling transport in neigh **(+)**<br>- walking recreation in neigh **(+)**<br>- cycling recreation in neigh **(-)**¥	added to the day's total number of steps<br>- pedometer data at least 4 days, including minimum 1 weekend day<br>- neighborhood was defined as " the direct environment, everywhere within a 10-15 min walk of your home"<br>- logarithmic transformations (log10) were used to improve normality of the PA variables<br>¥ trend towards significance, as p-value ranges between 0.05 and 0.10<br>- only variables "in neighborhood" and "whole week" are taken up for summary calculations
**Van Dyck et al.<br>2010**<br><br>**Ref n° 85**	- n = 1,166<br>- age range: 20-65y<br>- *M* age = 42.7y ±12.6<br>- 47.9% M	Clusters: purposeful<br>Individuals: random<br><br>- r.r. = 58.0%	BEPAS: 24 neighborhoods*[1] in Ghent, Belgium<br><br>Data collection May 2007-September 2008	CS	*Objective*:<br>(CSA accelerometers, model 7164))<br>**TOTAL PA**<br>- MVPA **= 1**<br><br>*Subjective*:<br>(IPAQ, long form last 7d)<br>**WALKING TRANSPORT**<br>- walking for transport **= 2**<br>**CYCLING TRANSPORT**<br>- cycling for transport **= 3**<br>**WALKING RECR**<br>- walking for recreation **= 4**<br>**LTPA**<br>- moderate LTPA **= 5**	*Subjective:*<br>(NEWS)<br>**RES DENSITY**<br>- residential density<br>**LAND USE MIX DIV**<br>- land use mix diversity<br>**CONNECTIVITY** - street network connectivity<br>**ACCESS SERVICES**<br>- land use mix access<br>- satisfaction with neighborhood services<br>**ACCESS RECR FACIL**<br>- convenience of recreation facilities (distance to!!)<br>**WALKING/CYCLING FACIL**<br>- availability and quality walking infrastructures<br>- availability and quality cycling infrastructures	<u>*Multivariate regression analyses*</u><br>- walkability*[2] **(+)**[1,2,3,4] (0)[5,6]<br>- street network connectivity **(+)**[3] **(-)**[4] (0)[1,2,5,6]<br>- availability and quality walking infrastructures (0)[1-6]<br>- availability and quality cycling infrastructures (0)[1-6]<br>- safety for cycling (0)[1-6]<br>- aesthetics **(-)**[1] (0)[2-6]<br>- safety from crime **(+)**[3] (0)[1,2,4,5,6]<br>- safety from traffic (0)[1-6]<br>- satisfaction neighborhood services **(-)**[2] **(0)**[1-6]<br>- convenience of recreation facilities **(-)**[3,6] (0)[1,2,4,5]<br><br>No sign moderation effects of gender	*[1] neighborhoods were stratified upon walkability and SES (4 different types)<br>- BEPAS = Belgian Environmental PA Study<br>- logarithmic transformations (log10) were used to improve normality of the PA variables<br>- all explanatory variables were centered on their means<br>*[2] PERCEIVED *walkability score* = z-score residential density + z-score land use mix diversity + z-score land use mix access<br>- 3 = only for high-SES adults<br>**Results**:<br>()[1] = related to MVPA<br>()[2] = related to walking transport<br>()[3] = related to cycling transport<br>()[4] = related to walking recreation

					- vigorous LTPA **= 6**	**SAFETY**<br>- safety for cycling<br>**TRAFFIC SAFETY**<br>- safety from traffic<br>**CRIME SAFETY**<br>- safety from crime<br>**AESTHETICS**<br>- aesthetics		()[5] = related to moderate LTPA<br>()[6] = related to vigorous LTPA
**Van Dyck et al.<br>2011<br><br>Ref n° 86**	- n = 412<br>- age range = 18y – 65y<br>-*M* age =48.745y ±11.99<br>- 51.9%F	Clusters: purposeful<br>Individuals: Random	12 high-walkable and 12 low-walkable neighborhoods in Ghent, Belgium<br><br><br>Data collection 2007-2008	CS	*Objective:*<br>**TOTAL PA**<br>7d accelerometer data<br>- MVPA<br>*Subjective:*<br>(IPAQ, long version last 7d)<br>**WALKING TRANSPORT**<br>- walking for transport<br>**CYCLING TRANSPORT**<br>- cycling for transport<br>**WALKING RECR**<br>- recreational walking<br>**LTPA**<br>- moderate-to-vigorous LTPA	*Objective:*<br>(GIS)<br>**WALKABILITY**<br>- walkability	*Independent sample t-tests*<br>Walkability characteristics are a prominent selection characteristic; more in women, older adults and lower educated<br>Difference between level of walkability in self-selection:<br>*No significant differences between low and high walkability*<br><br>*Multivariate regression analyses*<br>Multilevel!<br>*(adj for G, A and E)*<br><br>Total sample:<br>- walkability **(+)**[1,2,3] (0)[4,5]<br><br>Subsample high importance of walkability characteristics for self-selection neighborhood:<br>- walkability **(+)**[1,2,3] (0)[4,5]	- neighborhood self-selection was divided based upon the median (people above median were considered as those for whom walkability<br><br>**RESULTS**:<br>()[1]: specific results for (obj) **MVPA**<br>()[2]: specific results for (subj) **walking for transport**<br>()[3]: specific results for (subj) **cycling for transport**<br>()[4]: specific results for (subj) **recreational walking**<br>()[5]: specific results for (subj) **MVPA for recreation**
**Van Dyck et al.<br>2011<br><br>Ref n° 87**	- n = 3,500<br>- age range = 20y – 65y<br>- *M* age = 40.3y ± 12.9<br>- 53.5% F	Clusters: purposeful (in Ghent)<br>Individuals: random	59 neighborhoods in Ghent, Belgium	CS	*Subjective:*<br>(IPAQ short version, last 7d)<br>**TOTAL PA**<br>- MVPA	*Objective:*<br>(GIS)<br>**WALKABILITY**<br>- walkability<br>**RESIDENTIAL DENSITY**<br>- residential density per neighborhood	*Multilevel mediation modeling*<br>*Action theory tests*<br>*(adj for A, G, E and working status)*<br>- walkability **(+)**<br>- residential density per neighborhood **(+)**	- neighborhoods contain approximately 1,000 inhabitants each<br>- MVPA variable was logarithmically transformed to improve normality in the analyses
**van Lenthe et al.<br>2005**	- n = 8,767<br>- age range = 20y-70y	Random<br><br>- r.r. = 70.1%	GLOBE study:<br>78<br>neighborhoods in	CS	*Subjective:*<br>**ACTIVE TRANSPORT**<br>- walking/ cycling to	*Objective*: (opinions of professionals)<br>**ACCESS SERVICES**	*Multilevel logistic regression:*<br>*(adj for A, S, E and socioeconomic environment)*	*- cut off point for physical inactivity was 75min/wk<br>** dichotomized by "almost never"

**Ref n° 88**	- *M* age =<br>range= 45.2y ± 13.5 – 47.9y ± 14.6<br>- % gender range = 50.1% F – 51.9% F		Eindhoven, The Netherlands		shops/work*<br>**LTPA**<br>- walking / cycling / gardening leisure **<br>- participation in sports activities **<br><br>**! physical INACTIVITY!!**	- proximity to neighborhood facilities (food shops/sport and recreation facilities<br>**CRIME SAFETY**<br>- police attention required in neighborhood<br>**AESTH**<br>- general physical design neighborhood<br>- quality of green facilities in neighborhood<br>- amount of noise pollution from traffic in neighborhood	<u>Walking/cycling to shops/work:</u><br>- poor general physical design (0)[1,2]<br>- poor quality green facilities (0)[1,2]<br>- amount noise pollution **(-)**[1] (0)[2]<br>- poor proximity to food shops **(+)**[2] (0)[1]<br>- much police attention required (0)[1,2]<br><br><u>Walking/cycling /gardening leisure:</u><br>- poor general physical design **(+)** → *both 2nd and poor tertile*<br>- poor quality green facilities (0)<br>- amount noise pollution **(+)**<br>- much police attention required (0)<br><br><u>Participation sports activities:</u><br>- poor proximity to sports facilities **(+)**<br>- much police attention required **(+)**	and other categories<br>- neighborhood based on statistical units and have on average 2,200 residents<br>- general physical design, quality of green facilities and amount of noise pollution from traffic are proxies for "general attractiveness of the neighborhood"<br>- "police attention required" was a proxy for "safety in neighborhoods"<br>- proximity to food shops/ general physical design/quality green facilities/ noise pollution of traffic: *ref cat = good!!!*<br>- police attention required: ref cat = little<br>- tertiles were based on neighborhood scores, therefore, the n° of individuals in each tertile can differ.<br>**RESULTS**<br>()[1] specific results for participants aged 20y- 49y<br>()[2] specific results for participants aged older than 49y
**Van Tuyckom 2011**<br><br>**Ref n° 89**	- n = 24,846<br>- age range: ≥ 15y<br>- average % F EU:<br>36.1% F	- multi-stage random probabilistic	27 European Union member states (+ Bulgaria and Romania)<br><br>Data collection 2005	CS	*Subjective:*<br>(Eurobarometer survey)<br>**LTPA**<br>-LTPA	*Objective:*<br>**URBANIZATION**<br>"urbanization proxy measures"<br>- urban population %<br>- population density per km^2	<u>Bivariate linear regression analyses:</u><br>*OVERALL:*<br>- urban population % **(+)**<br>- population density per km^2 (0)<br><br>*MEN:*<br>- urban population % **(+)**<br>- population density per km^2 (0)<br>- paved roads (0)<br>- forest area in km^2 (0)<br><br>*WOMEN:*<br>- urban population % (0)	LTPA answer categories: "a lot", "some", "little" and "none"<br>LTPA was dichotomized into 2 categories: "not physically active in LT" (little and none) and "physically active in leisure time" (some and a lot)<br>- no data on vigorousness or duration<br>- urban population and population density were log transformed to achieve normality<br>- significance level 0.10

							- population density per km² (0)<br>- paved roads (0)<br>- forest area in km² (0)	
**Vandenbulcke et al. 2009**<br><br>**Ref n° 90**	- n = 3 924 299<br>- age range 18-65y<br>*M* age = 38.57 y old)<br>-42.96 %F	Working population (age range 18-65y)	589 communes in Belgium	CS	*Objective:*<br>(census data 2001)<br>**CYCLING TRANSPORT**<br>- proportion of bicycle use for commuting	*Objective:*<br>**ACCESS SERVICES**<br>- commuting distance (km)<br>**TRAFFIC SAFETY**<br>- accident risk (victims per 100,000min spent on bicycle)<br>**CRIME SAFETY**<br>- bicycle theft<br>- theft risk<br>**URBANIZATION**<br>- population density (inhabitants/km²)<br>- urban hierarchy (large city = H_1; small village = H_8)	*Unknown analysis*<br>- commuting distance **(-)**<br>→ >10 km: limit, independent of environment<br>→ ≤ 10 km: dependent<br>→ < 5 km: regional cities (H_2) are most popular for commuter cycling, large cities (H_1) least<br><br>*Correlations (Pearson):*<br>- commuting distance **(-)**<br>- dissatisfaction with cycling facilities **(-)**<br>- bicycle theft* **(+)**<br>- theft risk (0)<br>- accident risk* **(-)**<br>- urban hierarchy **(-)*[2]**<br>- population density **(+)**	- "accident risk": exposure to casualties was based on census data 2002-2005, only when the accidents required hospital treatment afterwards and were on weekdays!<br>- for urban hierarchy: largest cities (> 200,000 inhabitants) to smallest and least-populated communes (rural municipalities)<br>H_1= large cities; H_2 = regional cities; H_3 = small cities, well-equipped; H_4 = small cities, moderately equipped; H_5 = small cities, poorly equipped; H_6 = non-urban communes, well-equipped; H_7 = non-urban communes, moderately equipped; H_8 = non-urban communes, poorly equipped<br>*[1] = logarithmically transformed variable<br>*[2] Spearman correlation for urban hierarchy
**Vandenbulcke et al. 2011**<br><br>**Ref n° 91**	- n= 3 942 304<br>*M* age = 38.56 y old)<br>- 43.02 %F	Working population (no restriction on age range)	589 municipalities in Belgium	CS	*Objective:*<br>(census data)<br>- proportion of bicycle use for commuting	*Objective:*<br>**ACCESS SERVICES**<br>- commuting distance (km)<br>- short commute (%people who live at ≤10km from work)<br>**ACCESS RECR FACIL**<br>- recreational areas (% municipality)<br>**WALKING/CYCLING FACIL**<br>- dissatisfaction with cycling facilities<br>**TRAFFIC SAFETY**<br>- accident risk (victims per 100,000min spent on bicycle)<br>- traffic volume regional roads	*Bivariate correlations:*<br>- population density* **(+)**<br>- commuting distance **(-)**<br>- short commute* **(+)**<br>- town size** **(-)**<br>- urbanization* **(+)**<br>- recreational areas* **(+)**<br>- slope* **(-)**<br>- dissatisfaction with cycling facilities **(-)**<br>- bicycle theft* **(+)**<br>- theft risk (0)<br>- air pollution **(+)**<br>- accident risk* **(-)**<br>- traffic volume regional roads*	* logarithmically transformed variables

						- traffic volume<br>municipal/local roads **CRIME SAFETY**<br>- bicycle theft<br>- theft risk<br>**AESTH**<br>- air pollution<br>**URBANIZATION**<br>- population density (inhabitants/km²)<br>- town size (large city = 8; small village = 1)<br>- urbanization (% municipality that is urbanized)<br>**HILLINESS**<br>- slope (degree)	**(+)**<br>- traffic volume municipal/local roads* **(+)**	
**Wendel-Vos et al. 2004**<br><br>**Ref n° 92**	- n = 11,541<br>Males:<br>-n = 5353<br>-*M* age = 49.6y ± 10.5<br>Females:<br>-n = 6188<br>-*M* age = - mean age: 48.7y ± 10.9<br>- 53.6% F	Random<br>- r.r. = 66.4%	Maastricht, The Netherlands<br><br>Data collection 1998	CS	*Subjective:*<br>(SQUASH)<br>**WALKING RECR**<br>- time spent walking leisure<br>**WALKING TRANSPORT**<br>- time spent walking commuting<br>**CYCLING RECR**<br>- time spent bicycling leisure<br>**CYCLING TRANSPORT**<br>- time spent bicycling commuting	*Objective:*<br>(GIS)<br>**ACCESS RECR FACIL**<br>- green space area within 300m radius around postal code<br>- green space area within 500m radius around postal code<br>- recreational space*[1] within 300m radius around postal code<br>- recreational space*[1] within 500m radius around postal code	*Multilevel regression analysis*<br>*Crude analyses*<br>Total walking:<br>- woods (0)[1,2]<br>- parks (0)[1,2]<br>- sport grounds (0)[1,2]<br>- allotments (0)[1,2]<br>- day-trip grounds (0)[1,2]<br>Walking leisure:<br>- woods (0)[1,2]<br>- parks (0)[1,2]<br>- sport grounds **(+)**[2] (0)[1]<br>- allotments (0)[1,2]<br>- day-trip grounds (0)[1,2]<br>Walking commuting:<br>- woods (0)[1,2]<br>- parks (0)[1,2]<br>- sport grounds (0)[1,2]<br>- allotments (0)[1,2]<br>- day-trip grounds (0)[1,2]<br>Total cycling:<br>- woods (0)[1,2]<br>- parks (0)[1,2]<br>- sport grounds **(+)**[1] (0)[2]	- SQUASH= short questionnaire to assess health enhancing PA<br>- in the Netherlands, a six-position postal code represents on average 16.2 households<br>- neighborhoods were defined around the six-position postal codes: one with a 300m radius and one with a 500m radius<br>*[1] woods, parks, sport grounds (except for gymnasiums and fitness centers), allotments for vegetable gardens, and grounds for day trips (e.g. zoo, amusement parks)<br>**RESULTS**:<br>()[1] = related to 300m radius<br>()[2] = related to 500 m radius

							- allotments (0)[1,2]<br>- day-trip grounds (0)[1,2]<br><u>Bicycling leisure:</u><br>- woods (0)[1,2]<br>- parks (0)[1,2]<br>- sport grounds **(+)[1,2]**<br>- allotments (0)[1,2]<br>- day-trip grounds (0)[1,2]<br><u>Bicycling commuting:</u><br>- woods (0)[1,2]<br>- parks **(+)[1]** (0)[2]<br>- sport grounds **(+)[1]** (0)[2]<br>- allotments (0)[1,2]<br>- day-trip grounds (0)[1,2]	
**Wendel-Vos et al. 2008**<br><br>**Ref n° 93**	- n = 1,429<br>- age range: ≥18y<br>- 55% F	Clusters: Purposeful<br>Individuals: Random<br>- r.r. = 44%	15 neighborhoods in Amsterdam, The Netherlands<br><br>Data collection 2004	CS	*Subjective:*<br>(SQUASH)<br>**TOTAL WALKING**<br>- walking<br>**TOTAL CYCLING**<br>- cycling	*Subjective:*<br>**ACCESS PUBLIC TRANSP**<br>- accessibility of PT from home residence*[1]	*<u>Single level linear regression</u>*<br>*(adj for A, G, E and ethnicity)*<br><u>Walking</u>:<br>Model A → access PT (0)<br>Model C → access PT **(-)**<br><u>Cycling</u>:<br>Model A → access PT (0)<br>Model C → access PT **(-)**<br>*<u>Multilevel linear regression</u>*<br>*(adj for A, G, E and ethnicity)*<br><u>Walking</u>:<br>Model B→ access PT (0)<br>Model D → access PT (0)<br>Model E → access PT (0)<br><u>Cycling</u>:<br>Model B→ access PT (0)<br>Model D → access PT (0)<br>Model E → access PT (0)	- neighborhoods were defined with administrative boundaries!<br>*[1] item used for this perception is “my house is highly accessible by PT”; 5point scale, dummy coded (satisfied, not satisfied)<br>- men reported sign ↑cycling levels<br>- Model A = single level, individual<br>- Model B = multilevel, individual<br>- Model C = single level, contextual (not taking neighborhood into account)<br>- Model D = multilevel, contextual (taking between and within variance in neighborhood into account)<br>- Model E = multilevel, individual AND contextual<br>-for the summary calculations, only model A (=the least adjusted model) was taken up!

<u>Abbreviations:</u> F= female; *M* age= mean age; PA= physical activity; MPA = moderate physical activity; MVPA= moderate-to-vigorous physical activity; LTPA= leisure-time physical activity; r.r. = response rate; CS= cross-sectional; L= longitudinal; A = age (when adjusted for in the analysis); G = gender (when adjusted for in the analysis); E= education (when adjusted for in the analysis); M = marital status (when adjusted for in the analysis)
